# Experimental and Simulation Efforts in the Astrobiological Exploration of Exooceans

**DOI:** 10.1007/s11214-020-0635-5

**Published:** 2020-01-20

**Authors:** Ruth-Sophie Taubner, Karen Olsson-Francis, Steven D. Vance, Nisha K. Ramkissoon, Frank Postberg, Jean-Pierre de Vera, André Antunes, Eloi Camprubi Casas, Yasuhito Sekine, Lena Noack, Laura Barge, Jason Goodman, Mohamed Jebbar, Baptiste Journaux, Özgür Karatekin, Fabian Klenner, Elke Rabbow, Petra Rettberg, Tina Rückriemen-Bez, Joachim Saur, Takazo Shibuya, Krista M. Soderlund

**Affiliations:** 1 Archaea Biology and Ecogenomics Division, University of Vienna, Vienna, Austria; 2 Open University, Milton Keynes, UK; 3 NASA JPL, Pasadena, USA; 4 Freie Universität Berlin, Berlin, Germany; 5 German Aerospace Center (DLR), Berlin, Germany; 6 State Key Laboratory of Lunar and Planetary Sciences, Macau University of Science and Technology, Macau SAR, China; 7 Origins Center, Utrecht University, Utrecht, The Netherlands; 8 Tokyo Institute of Technology, Tokyo, Japan; 9 Wheaton College, Wheaton, USA; 10 Université de Bretagne Occidentale, Brest, France; 11 University of Washington, Seattle, USA; 12 Royal Observatory of Belgium, Brussels, Belgium; 13 German Aerospace Center (DLR), Cologne, Germany; 14 University of Cologne, Cologne, Germany; 15 Japan Agency for Marine-Earth Science and Technology (JAMSTEC), Yokosuka, Japan; 16 The University of Texas at Austin, Austin, USA

**Keywords:** Icy worlds, Techniques, Simulations, Experiments

## Abstract

The icy satellites of Jupiter and Saturn are perhaps the most promising places in the Solar System regarding habitability. However, the potential habitable environments are hidden underneath km-thick ice shells. The discovery of Enceladus’ plume by the Cassini mission has provided vital clues in our understanding of the processes occurring within the interior of exooceans. To interpret these data and to help configure instruments for future missions, controlled laboratory experiments and simulations are needed. This review aims to bring together studies and experimental designs from various scientific fields currently investigating the icy moons, including planetary sciences, chemistry, (micro-)biology, geology, glaciology, etc. This chapter provides an overview of successful *in situ*, *in silico*, and *in vitro* experiments, which explore different regions of interest on icy moons, i.e. a potential plume, surface, icy shell, water and brines, hydrothermal vents, and the rocky core.

## Introduction

The detection and characterization of Enceladus’ plume has been a major discovery of the Cassini mission (Hansen et al. 2006; Porco et al. 2006). The existence of a plume at Europa (Roth et al. 2014; Sparks et al. 2016, 2017; Jia et al. 2018) is promising for the future exploration by the NASA Europa Clipper mission. Enceladus’ plume brings its ocean into space, such providing unique information on an exoocean without the need of highly sophisticated drilling missions (Hedman et al. 2009; Hillier et al. 2007; Postberg et al. 2008; Waite et al. 2006; Postberg et al. 2009b, 2011b, 2018b; Waite et al. 2009, 2017; Hansen et al. 2011).

Space probes can be routed to fly through these plumes performing *in situ* investigations of the plume material, which is ejected from the interior oceans. However, to be able to interpret the data collected by instruments on board of missions, such as NASA’s Cassini or ESA’s JUICE (JUpiter ICy moons Explorer) probes, we have to conduct laboratory experiments to simulate the effects particles experience during such high-speed *in situ* sampling. For this purpose, both light gas guns (Grey et al. 2001; Harriss and Burchell 2017; Sheng-wei et al. 2017; Bowden et al. 2009; Judge 2017; Fisher et al. 2018; Fujishima et al. 2016; Lexow et al. 2013; Hibbert et al. 2017; Carver et al. 2008; Ramkissoon 2016) and laser induced liquid beam desorption (Postberg et al. 2009b, 2018b; Khawaja et al. 2019; Klenner et al. 2019, 2020a) can be used. The resulting atomic, molecular, and macroscopic fragments are then analysed using mass spectrometers. The inclusion of such spectrometers on board of future space missions (Kempf et al. 2012; Lunine et al. 2015; Reh et al. 2016; Mitri et al. 2018; Brockwell et al. 2016; Barabash et al. 2013) could help to detect potential characteristic patterns of biosignatures like amino acids, fatty acids, and peptides (Davila and McKay 2014; Creamer et al. 2017; Klenner et al. 2020a; Georgiou and Deamer 2014). At present, these *in situ* measurements are cost effective alternatives to sample return mission concepts.

Another possibility would be to include a lander or even a rover on a future space mission. This lander could be situated in the vicinity of a plume so that it could analyse particles that have been ejected by the plume and fell down on the surface. In order to simulate the conditions on the surface of an icy moon (extremely low temperature, high radiation, extremely low atmospheric pressure, etc.), simulation facilities on ground and simulation experiments conducted in the Low Earth Orbit (LEO), performed e.g. on the International Space Station (ISS), are used (Various Authors 2012, 2015; Rabbow et al. 2017). Further, icy terrestrial analogue sites like polar or high mountain regions have been investigated in this context (Garcia-Lopez and Cid 2017).

Even with the next generation of space missions, we will only receive an indirect insight into the interior of the icy moons by sampling either the plumes or the surface. Therefore, our understanding of the processes going on underneath the ice shell rely on laboratory or computer based experiments. These experiments include studies that examine material properties under subsurface conditions, e.g. the dynamics of ices, and high-pressure facilities used for both biotic (studies regarding microbial survival and biosignature production: Taubner et al. 2018, 2019; Foglia et al. 2016; Schuerger and Nicholson 2016; Hazael et al. 2017; Sharma et al. 2002) and abiotic settings (studies exploring ice-water and water-silicate interactions: Vance and Brown 2008, 2010, 2013; Mantegazzi et al. 2012, 2013). Further, investigations into environmental gradients are performed to broaden our understanding of the physical conditions on these icy worlds (More-Mutch et al. submitted).

Whether hydrothermal vents similar to those found on terrestrial ocean floors exist is under debate for some ocean worlds. The vents would add to the general habitability of the oceans by providing nutrients and an energy source. In addition to *in situ* studies of terrestrial hydrothermal vent fluids and ocean floor drilling (Beaulieu et al. 2015; Tivey 2011; Alt 2013), laboratory based experiments using large-scale reactors are conducted to reproduce closed-system water-rock interactions (Yoshizaki et al. 2009; Shibuya et al. 2013; Hsu et al. 2015; Sekine et al. 2015; Shibuya et al. 2015; Ueda et al. 2016), which are essential for our understanding of the ocean floor on these icy moons. Efforts are also being taken to develop microfluidics devices for a more controlled setting.

The majority of the experiments mentioned above rely on data obtained from computer-based simulations dealing with the interior structure of icy moons. These simulations use equations of states that help us to understand the mass distribution within a celestial body (Sotin and Tobie 2004; Iess et al. 2010; Van Hoolst et al. 2013). Further, computer-based simulations give an insight in the high-pressure phases of ice (Sotin and Tobie 2004; Sotin et al. 2007; Vance et al. 2014), silicates, and metals that may be formed within the subsurface, and can help visualise the interior dynamics like convection (Christensen 2015; Soderlund et al. 2014; Noir et al. 2009; Soderlund 2019; Journaux et al. 2019), assisting in the interpretation of the detected magnetic fields (Schilling et al. 2007; Kivelson et al. 2002; Saur et al. 2015; Hartkorn and Saur 2017; Iess et al. 2014).

To summarize, to gain a deeper understanding of the nature of icy moons, interdisciplinary work is essential, combining scientific fields like astronomy, geochemistry, (micro-)biology, etc. However, it is just as crucial to exchange techniques, models, and experimental designs. This review will give an overview about the various approaches used to gain a better picture about perhaps the most promising worlds in the Solar System to find extraterrestrial life.

## Plumes

### Plume and Sample Collection

The detection of plumes emanating from the surface of Europa and Enceladus indicates these worlds are active, and suggests the presence of subsurface oceans (Hansen et al. 2006; Porco et al. 2006; Roth et al. 2014; Sparks et al. 2016, 2017; Jia et al. 2018). These plumes provide valuable insight into the chemistry of the oceans and potential silicate interior of these icy moons. The Cassini spacecraft has provided us with a wealth of information regarding Enceladus, through the analysis of vapour and icy grains in the plume and the material making up Saturn’s E ring (Hedman et al. 2009; Hillier et al. 2007; Postberg et al. 2008; Waite et al. 2006; Postberg et al. 2009b, 2011b, 2018b; Waite et al. 2009, 2017; Hansen et al. 2011). Further information about both Europa and Enceladus could still be obtained through spectroscopy studies using terrestrial based telescopes, telescopes in Low Earth Orbit or spectrometers onboard orbiting spacecraft. For example, Roth et al. (2014) identified H and O emissions, in the southern hemisphere of Europa, using the Hubble Space Telescope’s Space Telescope Imaging Spectrograph (STIS). Additionally, sample collection missions, similar to NASA’s Stardust space probe (Brownlee 2003), would also provide further insight into the materials being ejected in the plumes of Enceladus and Europa, such missions have already been proposed by Neveu et al. (2014) and Tsou et al. (2014). The Stardust probe used a low density collection material, silica aerogel, to collect samples from the coma of comet 81P/Wild 2 and return them to Earth for analysis. The sampling tray consisted of rectangular blocks ($2\times 4~\mbox{cm}$) with thicknesses of 1 or 3 cm, within a frame that was wrapped in aluminium foil (100 μm thick); one side of the sampling tray was used to collect cometary material and the reverse side to collect interstellar material (Brownlee 2003).

Plume material venting from Enceladus are composed of a mixture of water ice, salts, silica particles, and organic material (Postberg et al. 2018a). The ejected material is thought to be travelling at velocities in the range of $80~\mbox{m}\,\mbox{s}^{-1}$ to $2~\mbox{km}\,\mbox{s}^{-1}$ (Hedman et al. 2009; Perry et al. 2016) and velocities between 7.5 to $17~\mbox{km}\,\mbox{s}^{-1}$ were experienced by the Cassini spacecraft as it passed through the plume (Postberg et al. 2018a). Therefore, laboratory based experiments simulating the ejection of material or sample collection conditions would be required to assist in the interpretation of data being collected. Light gas guns can be used to simulate, under controlled laboratory conditions, both the ejection of material from beneath a layer of ice and to simulate the hypervelocity impacts that would occur in a Stardust type sample return mission. Previous work utilising light gas guns and ice targets has examined the crater morphology of impacts into ice (Grey et al. 2001; Harriss and Burchell 2017; Sheng-wei et al. 2017) and have shown the survival of organic compounds in ejecta material when solid ice targets experience hypervelocity impacts (Bowden et al. 2009). However, using targets composed of a layer of ice over a liquid (which can be doped with potential organic compounds) could be used to generate a plume of liquid being ejected from beneath the ice. As plume material is ejected, infrared spectroscopy, for example, could be used to determine if theses organic compounds could be detected (Judge 2017). Investigations using a light gas gun to simulate a potential sample collection mission, have shown salt residue can still be detected after impact onto metal foils (Fisher et al. 2018) and the detection of peptides on silicate material after aerogel capture (Fujishima et al. 2016).

**Fig. 1:**
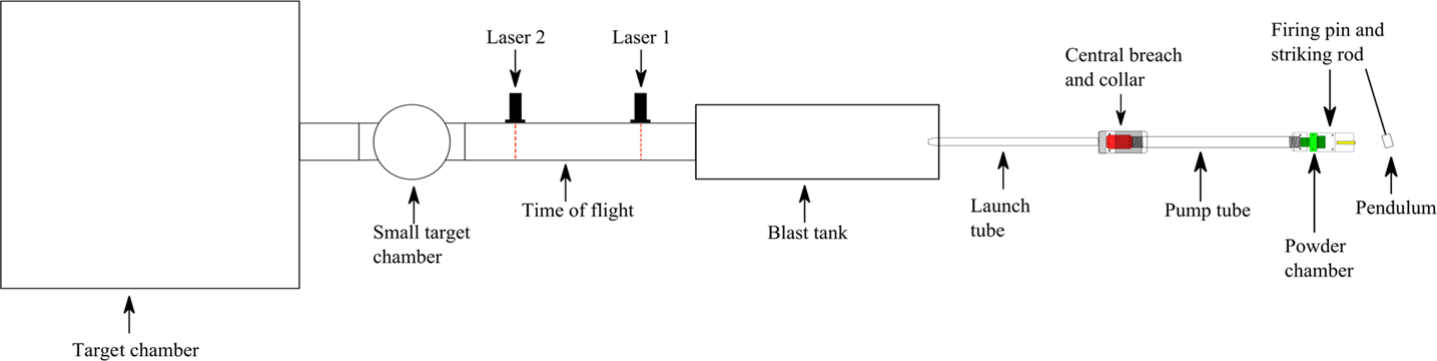
Schematic (not to scale) of a two stage light gas gun at the University of Kent (figure taken from Ramkissoon 2016)

Depending on the required impact velocity either a single stage (typically $<1~\mbox{km}\,\mbox{s}^{-1}$) or two stage light gas gun can be used in simulation experiments. Single stage light gas guns use a column of light gas (such as hydrogen, helium, nitrogen or krypton) to accelerate a projectile towards a target. Whereas, two stage light gas guns (Fig. 1) require the ignition of a propellant to push a piston, which compresses a column of light gas in two stage acceleration process. Once the light gas reaches a desired pressure (dependant on desired velocity) a burst disc ruptures allowing gas to expand into the launch tube accelerating the projectile towards the target. Two stage light gas guns are capable of accelerating projectiles at velocities up to $11.3~\mbox{km}\,\mbox{s}^{-1}$ (Lexow et al. 2013). However, it is possible to achieve velocities of ca. $100~\mbox{m}\,\mbox{s}^{-1}$ when using a single stage configuration (Hibbert et al. 2017). Projectiles range in sizes between millimetre to submillimetre size, and can be shot as either a single projectile or as multiple projectiles, known as a “buckshot”, for micron sized projectiles. However, larger light gas guns, such as Arnold Engineering Development Center (Carver et al. 2008), are able to fire projectiles up to 203 mm in diameter at slower velocities (ca. $4~\mbox{km}\,\mbox{s}^{-1}$). Projectiles made from a mixture of water ice and silicate material can also be fired in a light gas gun using a suitable “cold gun” configuration that will be the projectile frozen (Ramkissoon 2016), mimicking the interactions that would occur in a potential Stardust type sample return mission.

A cost effective alternative to sample return missions are mission concepts that analyse the plume material *in situ*. This was already successfully demonstrated with the Cassini instruments, most notably the Cosmic Dust Analyser (CDA, Srama et al. 2004), analysing the composition of individual plume ice grains, and the Ion and Neutral Mass Spectrometer (INMS, Waite et al. 2004), analysing the composition of the plume gases. These instruments were not limited by the small amount of vapour and ice grains present in the plume. They could characterise the plume material in detail and not only gave the first solid evidence for the existence of a subsurface ocean (Postberg et al. 2009b), but further constrained properties like temperature, salinity and pH, and also the organic material emerging from the subsurface ocean (Postberg et al. 2008, 2009b, 2011b; Glein et al. 2015; Hsu et al. 2015; Postberg et al. 2018b). Currently several mission concepts with an advanced *in situ* payload that builds upon the successful Cassini instruments have been developed (Lunine et al. 2015; Reh et al. 2016; Mitri et al. 2018).

### Lab Experiments for the *In Situ* Detection of Abiotic and Biotic Organics in Ice Grains from Ocean Worlds

Mass spectrometers on board Cassini have been proven to be the most effective instruments to characterise the composition of plume material. Investigations by the CDA and the INMS delivered crucial data to infer chemical and physical properties of Enceladus’ subsurface ocean and render the Enceladus ocean to be the best characterised extraterrestrial water environment (Spahn et al. 2006; Waite et al. 2006, 2009, 2017; Postberg et al. 2008, 2009a,b, 2011b, 2018b; Khawaja et al. 2019).

Of particular interest is the organic material in the plume and the question if this organic material might be of biogenic, prebiotic or primordial origin. To distinguish these possibilities it is important to understand the mass spectral appearance of biotically and abiotically produced organics inside ice grains from the Enceladean plume in spaceborne mass spectrometers. Here, we give an overview of a laboratory method to simulate mass spectra of organic bearing ice grains that hit detectors onboard spacecraft flying by Enceladus and also other potentially ice grain emitting ocean bearing moons like Europa. The method is not only applicable to ice grains from a plume as it is apparent on Enceladus and possibly on Europa (Roth et al. 2014; Sparks et al. 2016, 2017) but is also capable to simulate the composition of any water dominated icy grain hitting a mass spectrometer at hypervelocities ($>1~\mbox{km}\,\mbox{s}^{-1}$) in space. Examples would be cometary ice grains or ejecta lifted from the surface of icy moons by impacts of micro-meteoroids (Kruger et al. 1999; Postberg et al. 2011a; Kempf et al. 2012; Paganini et al. 2019). The experiment is currently optimised for impact ionisation detectors like the CDA, the SUrface Dust Analyser (SUDA) (Kempf et al. 2012) or the ENceladus Ice Analyzer (ENIA) (Lunine et al. 2015; Reh et al. 2016; Mitri et al. 2018) but in the near future will be capable to simulate spectra of ice grains as they will appear in neutral gas mass spectrometers like INMS (Waite et al. 2004), MASPEX (Brockwell et al. 2016), or PEP-NIM (Barabash et al. 2013).

**Fig. 2:**
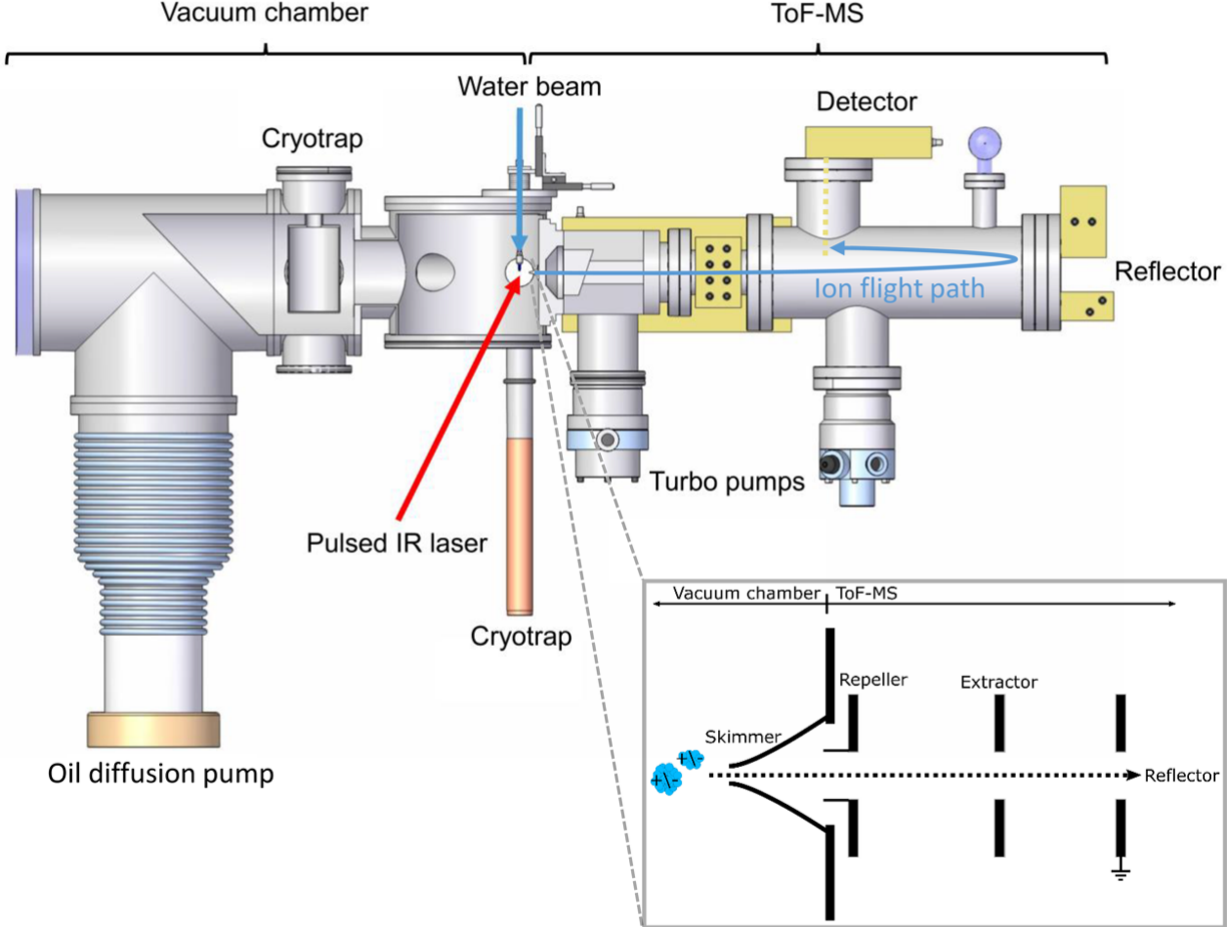
Laboratory LILBID-TOF-MS apparatus for simulating hypervelocity ice grain impacts onto impact ionisation mass spectrometers in space. The instrument configuration is shown in the schematic on the bottom right (adapted from Postberg et al. 2018b)

The method of Laser Induced Liquid Beam Ion Desorption (LILBID) has been shown to be a vital tool for simulating hypervelocity impacts of ice grains onto impact ionisation mass spectrometers in space (Postberg et al. 2009b, 2018b; Khawaja et al. 2019; Klenner et al. 2019). The experimental setup (Fig. 2) is described in detail in Klenner et al. (2019), and as such we provide a brief overview here. A water beam (radius of 6 μm–10 μm) with dissolved or dispersed analytes is injected into a high vacuum ($5 \times 10^{-5}~\mbox{mbar}$) and irradiated with a pulsed infrared laser (20 Hz, 7 ns pulse length). The laser (with adjustable laser power densities) operates at a wavelength of approx. 2850 nm to match the absorption frequency of the OH-stretch vibration of water. The water beam absorbs the laser energy and explosively disperses into atomic, molecular and macroscopic fragments thereby simulating a hypervelocity impact of an ice grain. As in space, cations, anions, electrons, and neutral molecules are created (Klenner et al. 2019). Cations and anions are analysed in a reflectron type Time-of-Flight mass spectrometer (TOF-MS) with its mass resolution of about $600\mbox{--}800~\mbox{m}/\Delta \mbox{m}$. The mass spectrometer uses the principle of delayed extraction (Klenner et al. 2019). With delayed extraction, ions as a function of their initial velocity are selected by setting a delay time for this gating system. By variation of both the lasers power density and the delay time of extraction, impacts of ice grains onto space detectors with speeds ranging from approx. $1\;\mbox{to}\;20~\mbox{km}\,\mbox{s}^{-1}$ can be simulated (Klenner et al. 2019).

Some of the most widely accepted potential biosignatures of water-based organic life forms applicable to extraterrestrial ocean worlds are amino acids, fatty acids, and peptides. Amino acids and fatty acids of course can be formed by abiotic process, too.

For example amino acids found in meteorites (Cronin and Pizzarello 1983) are produced abiotically (Ménez et al. 2018). However, the simplest amino acid glycine (Gly) is excessively abundant compared to other amino acids (Higgs and Pudritz 2009), because abiotic amino acid synthesis follows the rules of thermodynamics. In biotically modified systems, more complex amino acids than Gly become prevalent. As a consequence, the abundance pattern of amino acids, i.e. the ratio of various amino acids to Gly, can be considered as a biosignature (Davila and McKay 2014; Creamer et al. 2017; Klenner et al. 2020a; Taubner et al. 2019).

Fatty acids are commonly found in the lipid bilayer membranes of known life forms and can, therefore, also serve as a biosignature. In an abiotic Fischer-Tropsch synthesis fatty acids are produced by the catalysed addition of single carbon atoms. The abiotic fatty acid abundance pattern is not selective and no obvious abundance pattern in chain length and carbon number emerges (McCollom and Seewald 2007). In contrast, fatty acids from a bacterial source show an obvious abundance pattern in which even carbon number fatty acids clearly dominate over odd carbon number fatty acids because carbon atoms are added two at a time during biosynthesis (Georgiou and Deamer 2014). Moreover, the majority of hydrocarbon chains that are well able to assemble into stable bilayer membranes have lengths of 14 to 20 carbon atoms (Georgiou and Deamer 2014).

In contrast to amino acids and fatty acids, peptides (oligomers or polymers consisting of two or more amino acid residues) may directly indicate active biochemistry because the generation of these complex molecules must have exceeded their decomposition by different mechanisms like hydrolysis.

**Fig. 3:**
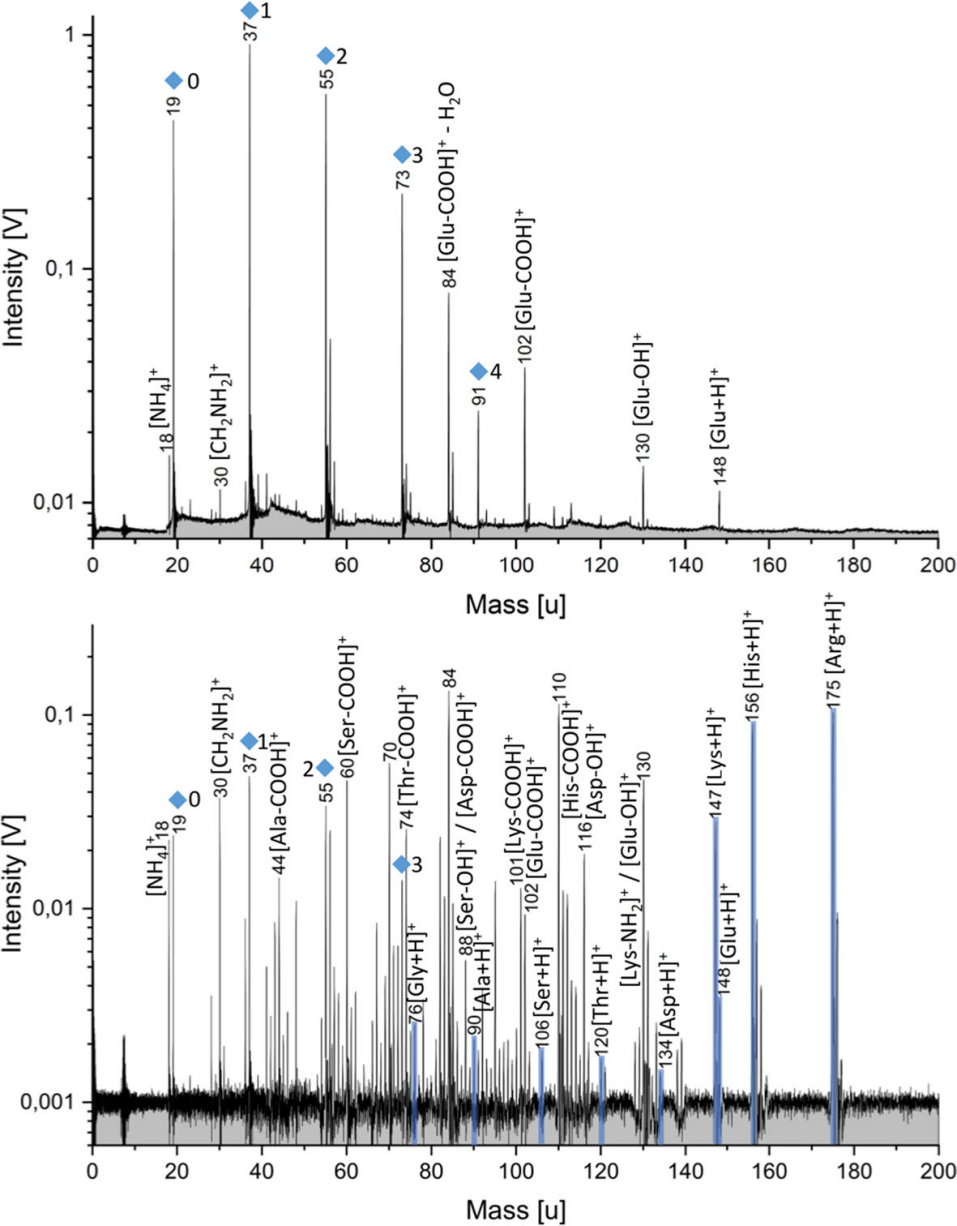
Top: Laboratory mass spectrum (y-axis in logarithmic scale) of 50 ppmw Glu in the cation mode of the mass spectrometer. Glu fragments due to the loss of OH and COOH are observed. Glu–COOH additionally loses H_2_O (observed at m/z 84). Bottom: Laboratory mass spectrum (y-axis in logarithmic scale) of nine amino acids (50 ppmw each) dissolved together in the positive detection mode. The protonated molecular peaks are highlighted in blue. Peaks, with varying intensities, corresponding to each amino acid can be detected. Mass peaks from the water matrix of the form (H_2_O)_*n*_H_3_O^+^ are marked by blue diamonds (figure taken from Klenner et al. 2020a)

With the LILBID setup, the mass spectra of water ice grains carrying trace amounts of these organic substances encountered by a spaceborne detector can be simulated. Furthermore, biotic and abiotic signatures can be mimicked and detection limits for the instruments in space can be predicted. Figure 3 shows cationic laboratory mass spectra of 50 ppmw glutamic acid (top) and of a variety of amino acids, each at a concentration of 50 ppmw (bottom), dissolved in water (Klenner et al. 2020a). The protonated molecular peaks and certain characteristic fragments are easily detectable. However, the varying amplitudes of the different amino acids (Fig. 3 bottom) indicate varying sensitivities dependent on the tendency of the species to form cations (Klenner et al. 2020a).

**Fig. 4:**
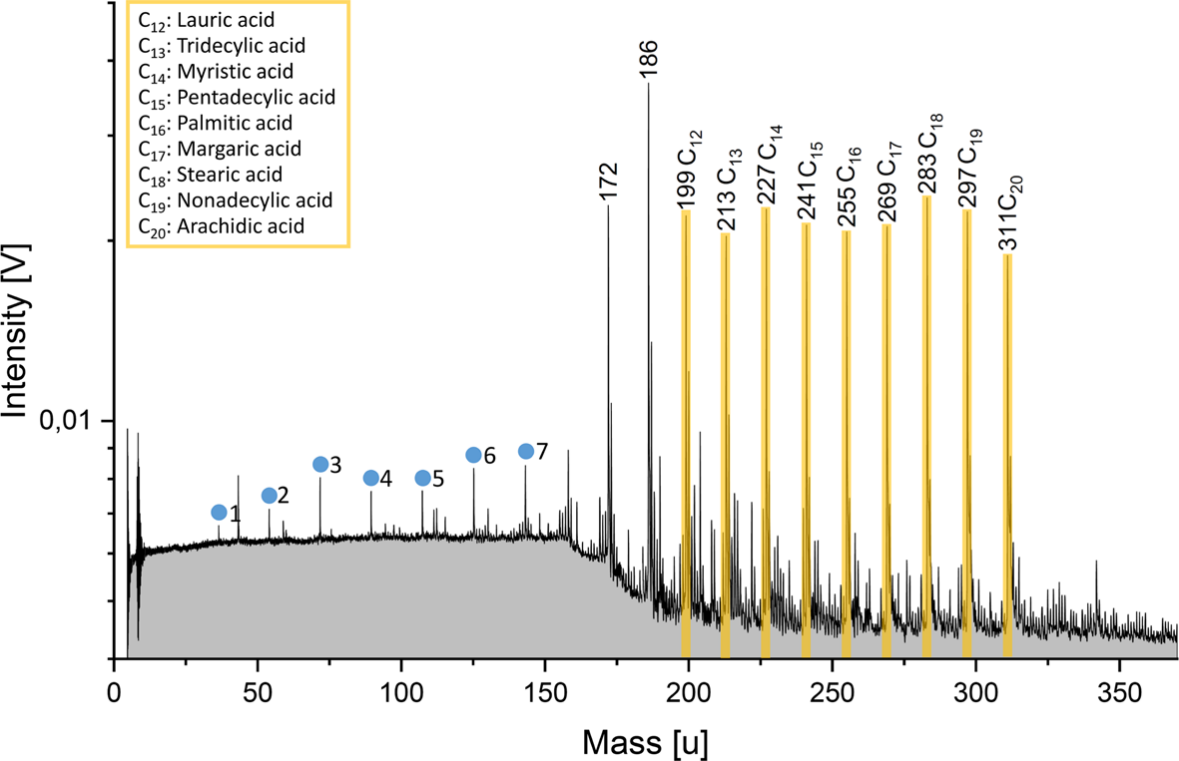
Anion mass spectrum (y-axis in logarithmic scale) of fatty acids at equal concentrations of $5.5\times 10^{-6}~\mbox{M}$ in a water-acetonitrile matrix (50/50 vol.-%) simulating ice grain impact velocities of $4\mbox{--}5~\mbox{km}\,\mbox{s}^{-1}$. The respective carbon numbers of the fatty acid molecules are labelled above the peaks. Deprotonated molecular peak intensities (yellow) are approximately equal, consistent with the equal fatty acid concentrations. Peaks of the form [(H_2_O)_*n*_OH]^−^ are observable due to the watery matrix (blue circles). The respective $n$ is labelled besides the peaks (figure taken from Klenner et al. 2020a)

In contrast to amino acids, fatty acids are best observable in their anionic form in mass spectra. The anion spectrum of a mixture of fatty acids with carbon numbers from 12 to 20 is shown in Fig. 4. In this case, the fatty acids are dissolved in a water-acetonitrile solution (50/50 vol.-%) because of their poor solubilities in pure water. Deprotonated molecular fatty acid anions [M-H]^−^ are observable. The molecular peaks are equally high, consistent with the equal fatty acid concentrations of $5.5 \times10^{-6}~\mbox{M}$. Detection limits of individual amino acids and individual fatty acids in the LILBID facility are found to be on the ppb level (Klenner et al. 2020a).

These measurements are part of a comprehensive spectral reference library for *in situ* mass spectrometers in space from a wide variety of organic analogue materials in icy grains and become a template for past and future space missions. Results of another study about the analysis of mass spectra of biosignatures in a more complex matrix are underway (Klenner et al. 2020b). In this study, salts and other organic substances are added to amino acids and fatty acids and biotic and abiotic fingerprints of these biosignatures are discriminated.

## Surface

The realisation of future space exploration missions is challenging as the main drivers to realise such missions are scientific questions based on our understanding of the habitability of planets and moons as well as on the task to detect life in extraterrestrial environments. To improve the scientific interpretation of data obtained from these future exploration missions, merging data obtained from planetary analogue studies (Garcia-Lopez and Cid 2017) with ones obtained in planetary simulation laboratories and in planetary simulations directly in space on space exposure facilities will be the main task to be pursued.

Caused by the far distances between Earth and the other planets in the Solar System, missions to the scientifically interesting planetary objects are rare and need on average about 10 years of preparation before launch. Therefore, planetary analogue field site investigations are fundamental to start mission preparations. Taken into account that no terrestrial analogue side will perfectly mimic conditions on the icy moons, the best approach are icy environments in the terrestrial polar or high mountain regions. There, studies on the ice itself, the border line between solid and liquid water and the potential habitats for life as well as life detection tests are possible. Glaciers above subglacial lakes or above the sea including thick sea ice are suggested to be the best objects for study. Landers or rovers equipped with specific exploration hardware such e.g. a melting probe could be tested on and in ice even to be used below the ice for investigation of the lake or sea water. According to the instruments it is necessary to use hardware to be able to detect organics and organisms and even to collect these samples. The organic and biological samples could hereafter be used for further studies investigating the habitability of icy environments as well as for the habitability of the icy ocean moons in specific planetary simulation chambers or on exposure platforms in space.

In addition to the analysis of the ice and the liquid water phase and their ingredients other test locations are needed. It is necessary to investigate crevasses and ice caves as well as particular hydrothermal to volcanic active areas, which would much approach the conditions nearby the plumes which were observed on the icy moons (Hsu et al. 2015; Kattenhorn and Prockter 2014; Waite et al. 2009). Samples collected from these areas will give information about how to operate life detection work and the sample collection in such extreme active environments.

An important step for understanding the habitability of the icy ocean worlds in the outer Solar System is to test collected samples from the planetary analogue field sites mentioned above under simulated conditions approaching as much as possible the observed conditions of the icy moons. Apart from Titan, ocean worlds of the Solar System are too small to stabilise any atmosphere worth mentioning. Pressures close to the ice surface in general is similar to that in LEO, probably with regional differences. Temperatures are close to space temperature. As determined from Hubble data, the European atmosphere is composed mainly of oxygen at a pressure of $1\times 10^{-6}~\mbox{Pa}$ and surface temperatures are 110 K near the equator and 50 K at the poles. Liquid water is most likely not available. While active growth of organisms is very unlikely, low temperatures increase survival rates of most organisms. In contrast, the low pressure, the high radiation as well as the full spectrum solar UV radiation not attenuated by atmospheric interaction are detrimental to organisms (Horneck et al. 1996). Simulation of the surface conditions are performed in temperature controlled, cooled vacuum facilities equipped with solar simulators capable to simulate the short wavelength UV. Additional radiation sources to simulate the cosmic radiation as X-ray in combination with the low pressure provide more realistic surface environment simulation conditions for the icy moons. Temperature at sample site in vacuum should be as low as possible, and maintained below 273 K, to simulate the continuous frozen state of dry samples. While active growth is not possible due to lack of liquid water in vacuum, survival in a passive form is investigated under these simulation conditions.

If the stability of organics or even organisms could be observed in and on the simulated analogue ice compared to the simulated ocean, life detection locations could be postulated and distinct life detection operation scenarios could be elaborated for future missions. If organisms are able to survive or even to be active under the simulated planetary simulation conditions, a clear statement on the habitability of the simulated world could be postulated.

Besides of that also the probes and their technical equipment must be tested in conditions which are very close to the conditions on the icy moons. The above mentioned extreme temperature regimes in addition to vacuum and extreme radiation could also exert a significant impact on the technological systems.

The last step to realise the best approach to simulate and test the conditions of the icy ocean worlds is performing simulation experiments directly in space. Exposure experiments of organics and biomolecules relevant and adapted to aquatic and icy environments such as those selected in terrestrial planetary analogue environments will give information about the potential of their stability and their potential to be detectable after being exposed to all extreme space conditions. First exposure experiments have been performed in LEO, simulating e.g. the conditions on Mars and analysing the stability and detectability of biomolecules after being exposed to space (de Vera et al. 2012; Leuko et al. 2017) as well as survival of different organisms. Similar experiments are needed now in reference to the icy ocean worlds:

1. Passive exposure experiments in space, e.g. on the ISS, utilise the complex space radiation environment as reference of the high and complex surface radiation environment on the icy ocean worlds combined with the low pressure in orbit (varying between $1.33\times10^{-3}$ and $1.33\times10^{-4}~\mbox{Pa}$ as determined by the Russian agency RSC Energia for the outside ESA exposure facility EXPOSE-R2, depending on the direction with respect to flight direction) to provide data on the radiation and vacuum stability of biomolecules and resistance of organisms to ocean world surface conditions.
2. Active life forms depend on the availability of liquid water, an energy source and at least basic nutrition. Features like the tiger stripes and linea of the icy ocean world surfaces give hints on a possible turnover of surface material and ocean material as well as possible niches harbouring liquid water, most likely in form of high salt brines, near the surface in addition to the oceans. The ability of organisms to actively metabolise and grow in high salt water at low temperature with at least partly exposure to the surface radiation including the unshielded solar short UV radiation are planned to be investigated in new developed ESA space exposure facilities “EXPO” on the outside Bartolomeo platform at the European Columbus module of the ISS. The Science Modules 1 for the experiments IceCold and ExoCube allow the *in situ* monitoring of active microbial cultures in the LEO radiation environments while exposed to extraterrestrial solar UV and after exposure respectively. The Science Module 2 within EXPO contains hardware developed for the space experiment BioSigN and allows exposure of selected ocean relevant microorganisms, organics and potential biosignatures. The experiment has a similar setup to the previous experiments in the EXPOSE-R2 with BIOMEX (de Vera et al. 2012) so that direct comparison with previously performed space experiments is possible and research on the stability of biomolecules with relevance to icy ocean worlds can be realized. EXPO and the Science Modules 1 and 2 are currently developed by ESA. Similar experiments are highly needed to better understand the possibility of life to exist or even thrive under the expected icy ocean world environmental conditions.

The passive exposure space experiments have been performed recently in three international missions on the ESA multiuser exposure facility EXPOSE on ISS. Organisms and biomolecules were exposed to LEO and Mars pressure and atmosphere, temperature regimes between 274 and 323 K, galactic cosmic rays and extraterrestrial solar UV to investigate their stability, resistance and survival (Various Authors 2012, 2015; Rabbow et al. 2017). Additional passive space exposure experiments are currently planned for the ESA EXPO Science Modules 2, focusing amongst others on the response of anaerobic life forms to these conditions.

## Ice/Water Interactions

Improving the theoretical basis for investigating habitability in icy ocean worlds requires further study of the material properties under conditions occurring in icy ocean worlds. In the largest of the Solar System’s ocean worlds, Ganymede, pressures at the silicate-water interface are around 1.6 GPa (16,000 bar) (Vance et al. 2018). Temperatures can run the full range of liquid stability. Given the likely presence of low-freezing-point volatiles such as ammonia beyond Jupiter, the lowest temperatures in extraterrestrial oceans could approach 200 K. Experiments in recent decades have investigated material properties under these conditions. Important for understanding the dynamics of ices (phases Ih, III, V, and VI) is having constraints on the rheology (Durham et al. 1997, 2010) and thermal properties (Andersson and Inaba 2005). These properties control retention of heat in icy ocean worlds, including the onset and vigor of solid state convection. The stability of different phases and their interaction with impurities are also important (Journaux et al. 2013, 2017). The thermodynamics of aqueous solutions are investigated using piston-cylinder (Vance and Brown 2008, 2010, 2013) and diamond-anvil systems (Mantegazzi et al. 2012, 2013). The theoretical basis for using these data has improved, allowing for more intuitive derivation of equations of state (Brown 2018). Freely available software for minimising the Gibbs or Helmholtz energy of multi-component systems are under continued development (Connolly 2009; Lemmon et al. 2010), and are increasingly being used for the study of realistic interior structure and dynamics (Neveu et al. 2015; Vance et al. 2018).

## Subsurface Water/Brines in the Context of Microbial Studies

### High Pressure Simulation Facilities

High pressure simulation facilities are used to simulate the conditions in the sub-surface oceans and to determine the products of silicate-water interactions.

**Fig. 5:**
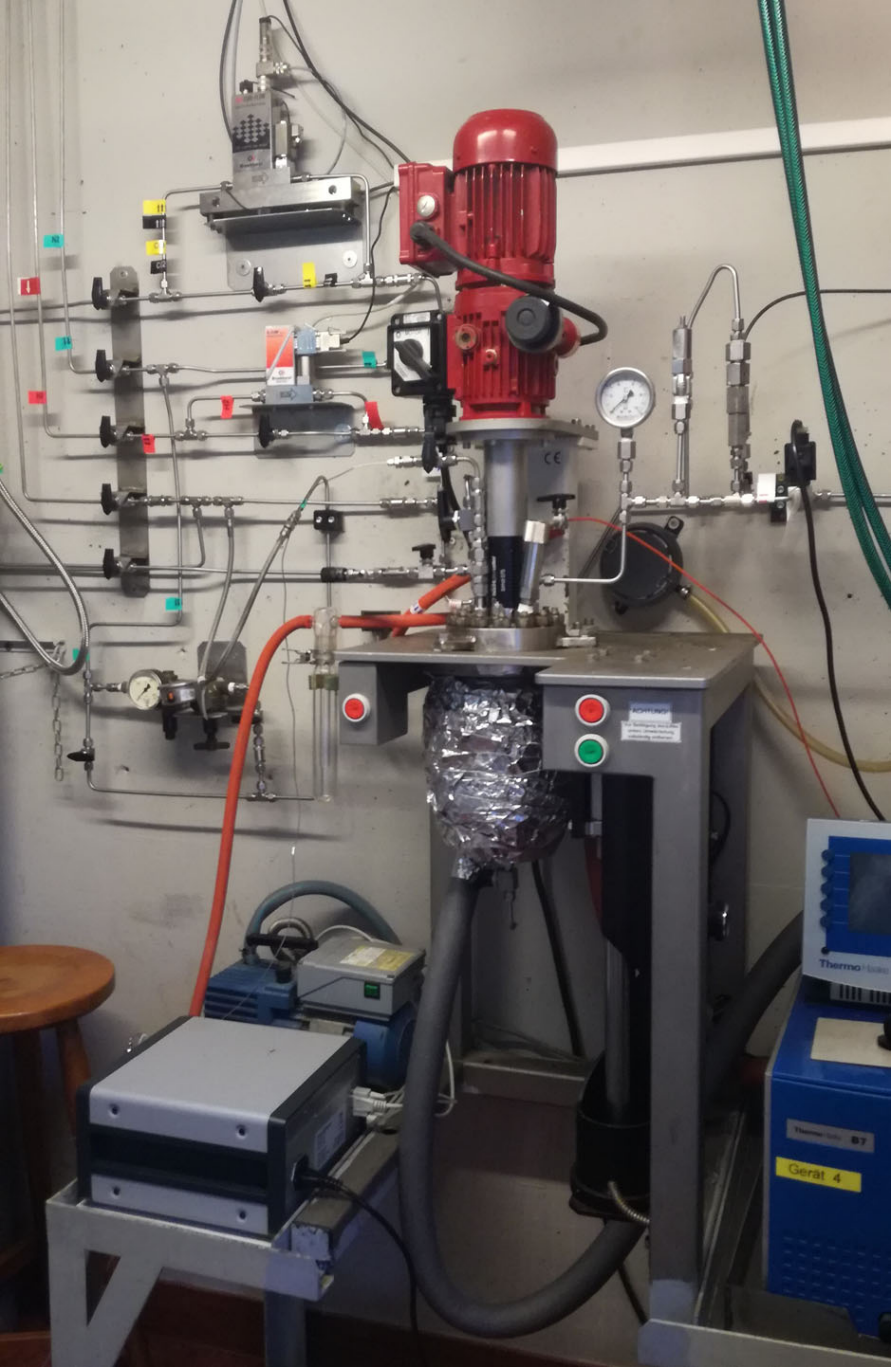
High-pressure reactor system at the JKU Linz (Credits: Patricia Pappenreiter, JKU Linz)

To investigate the potential of habitability in a sub-surface ocean, laboratory based simulation experiments are required. Work has predominantly focused on the compositional effect of the ocean on microbial growth (e.g., Stevens et al. 2018; Avendaño et al. 2015; Fox-Powell et al. 2016). Although the environmental parameters within the sub-surface oceans of Europa (e.g., Kargel et al. 2000; Zolotov and Kargel 2009) and Enceladus (e.g., Glein and Shock 2010; Zolotov 2007; Postberg et al. 2009b, 2011b; Iess et al. 2014; Hsu et al. 2015) have been modelled, little is known if microorganisms could grow under such conditions. This is predominately due to the difficultly in simulating the relatively high pressures associated with these environments. For example, when investigating the effect of Mars conditions on microbial growth, experiments are routinely conducted in glass containers (e.g., Schirmack et al. 2015; Mickol et al. 2018), which are unable to sustain pressures above 1 bar. To alleviate this issue, high pressure reactors are used. For example, Taubner et al. (2018) investigate the feasibility of methanogenic growth under simulated Enceladus conditions using a stainless steel Büchi reactor (Fig. 5), which was continuously stirred. The growth experiments were carried out over a range of pressure up to 90 bar and the experiments were performed with either a H_2_/CO_2_ (4:1) gas phase, a H_2_/CO_2_/N_2_ (4:1:5), or, as in the final experiments, with a N_2_/H_2_/CO_2_/CO/C_2_H_4_ (32.5:55:6.5:3:3) mixture.

At higher pressures above 1000 bar, microbial survival is investigated using a diamond anvil (e.g., Foglia et al. 2016; Schuerger and Nicholson 2016; Hazael et al. 2017). For example, the effect of high pressure (0.0006–0.01 Mbar) on the physiology and metabolic activity of *Shewanella oneidensis* and *Escherichia coli* was investigated (Sharma et al. 2002). A diamond anvil consists of a sample chamber located between two diamonds, which when squeezed exerts high static pressure on the sample. Continuous monitoring is possible through the diamonds, using a suit of analytical techniques, such as X-ray Raman, X-ray diffraction and Mössbauer spectroscopy (Bassett 2009).

The geochemical interaction between the silicate-based core and the ocean brine could be a potential source of energy and bio-essential elements for microbial life. Serpentinisation, which produces molecular hydrogen, which is a potential energy source for life, has been investigated at temperatures below 373 K using ultramafic rocks and the mineral olivine with aqueous solutions (e.g., Stevens and McKinley 2000; Neubeck et al. 2011; Mayhew et al. 2013; Okland et al. 2014; McCollom and Donaldson 2016). Routinely, minerals substrates are heated and stored in rubber stoppered glass bottles and the production of hydrogen, methane and silicon dioxide is measured. However, there are limitations to these studies due to the high levels of hydrogen, methane and silicon dioxide released during heating, which are produced by the reactor materials (McCollom and Donaldson 2016; Barge and White 2017).

In terrestrial environments, silicate-based interactions are also a source of bio-essential elements for microbial life. Studying silicate-based interactions on Earth can be done by using either closed-systems, for example batch experiments, or open-systems, for example dialysis-cell reactors and continues-flow reactors. Although simulation facilities have been developed to investigate the habitability of life within hydrothermal vents on the icy moons, there have been limited studies, which have focused on the silicate-water reactions at the ocean floor of icy oceans. A high pressure flow-through facility has recently been developed, which allows simulated ocean brine to be continuously circulated through silicate material (Hamp et al. 2018; Ramkissoon et al. 2018). Using a high-performance peristaltic pump, the brine is pumped through into a pressure vessel containing silicate material. The temperature, pressure and gas composition within the chamber are automatically controlled to simulate the required conditions (maximum pressure 200 bar and temperature range from 263 to 573 K). Samples are collected from the outflow for analysis. Microorganisms can be grown within the high pressure chamber as the flow rate can be altered to allow steady-state microbial growth.

Besides brines, also clathrates are investigated from the perspective of icy moon research. Clathrate hydrates are inclusion compounds in which small guest molecules (i.e., CH_4_, CO_2_, etc.) are trapped inside highly symmetric water cages. These ice-like crystalline solids are abundant on Earth under high-pressure conditions, primarily existing in the permafrost and marine sediments. Their existence elsewhere in the Solar System, although predicted by models, remains to be demonstrated. Icy celestial bodies whose pressure/temperature conditions on the surface and the interior are favourable to the formation of gas hydrates are also expected to contain substantial amounts of these materials. A high-pressure apparatus, which consists of a liquid N_2_-cooled cryostage equipped with a flow-pressure capillary tube, has been developed exclusively for studying the methane clathrate formation and its exchange kinetics (Vu and Choukroun 2015). The maximum allowable working pressure inside the capillary tube is 200 bar. Methane diffusion at the interface of clathrate hydrate structures is relevant for the methane mobility in methane hydrates embedded in the cryosphere of large icy bodies. This has been studied by Ranieri et al. (2017) using quasielastic neutron scattering (QENS) technique with pressures up to 8000 bar (0.8 GPa).

The existence of clathrate hydrates in solid bodies of the Solar System is very hard to verify and most of the available information is based on limited experiments and theoretical models. The unequivocal confirmation of clathrate hydrate detection requires spectroscopic means. The spectrometric signatures of clathrates have been studied experimentally for identification in near to mid-infrared wavelengths using space-based observations or *in situ* probes (Dartois and Schmitt 2009; Dartois et al. 2010). For this purpose a dedicated evacuable enclosed cell was built to study the signatures at low temperature, high-vacuum, ($P< 10^{-7}~\mbox{mbar}$). Presence of clathrate hydrates in conditions to analogous interstellar medium (extreme high vacuum and ultracold conditions) has been suggested by the experiments conducted in an ultrahigh vacuum instrument (base pressure approx. $10^{-10}~\mbox{mbar}$) equipped with RAIR spectroscopy and TPD mass spectrometry (Ghosh et al. 2019).

### Laboratorial Replication of Environmental Gradients for Microbiological and Astrobiological Studies

A relevant question regarding simulation experiments is the need to develop better suited systems which allow us to better mimic natural gradients in the lab and are also compatible with isolation and cultivation of microbial strains. The natural habitats of most microbes are extremely dynamic and rich in spatial gradients of e.g., growth substrates, electron acceptors, pH, salts, and inhibitory compounds (e.g., Brune et al. 2000; Emerson et al. 1994). Such gradient-rich conditions are particularly marked in several terrestrial analogue sites, and expectable in the exooceans of the icy moons of our Solar System.

Despite this well-known documented fact, most microbiology isolation and cultivation efforts remain heavily dependent on the classical methods, with reliance on agar-based homogeneous media that share little resemblance with the complexity of the natural environment. The advent and widespread use of molecular phylogenetic studies together with other culture-independent studies revealed the inadequacy of such standard cultivation methods to provide us a clear and faithful picture of the existing microbial biodiversity. Indeed, current estimates point to more than 99% of the microorganisms observable in nature evading cultivation (e.g., Oren 2004; Schleifer 2004), which highlights short-comings in the laboratorial replication of natural conditions and subsequent bias towards certain phylogenetic groups (Overmann et al. 2017). Such limitations are perceived as a significant bottleneck in the fields of Microbiology and Astrobiology.

The use of agar-based solid media remains the most popular microbial enrichment, isolation, and cultivation strategy, partly due to its low-cost and ease of preparation and use. Attempts to further extend the applicability of such media, namely by the development of plate diffusion methodologies, have been limited and remain under-explored.

Plate diffusion methodologies owe much to the pioneer works of Beijerinck, and later works that resulted in the development of the ingenious wedge plate technique by Szybalski and Bryson (Szybalski 1951; Szybalski and Bryson 1952) and the steady-state two-dimensional gradient plate (Caldwell and Hirsch 1973), which constituted two major breakthroughs in this field. Further studies by Wimpenny and colleagues (Peters et al. 1991; Thomas and Wimpenny 1993, 1996b,a; Wimpenny and Waters 1984, 1987; Wimpenny et al. 1981) perfected the wedge plate technique and even permitted the establishment of multidimensional systems (i.e., systems where several parameters are varied simultaneously). Despite such advances, the full potential of plate diffusion methods in the several fields of microbiology has remained unexplored, with its applications remaining almost exclusively restricted to auxanographic studies (for a review on the evolution and applications of plate diffusion methods, see Wimpenny and Jones 1988; Wimpenny 1988).

**Fig. 6:**
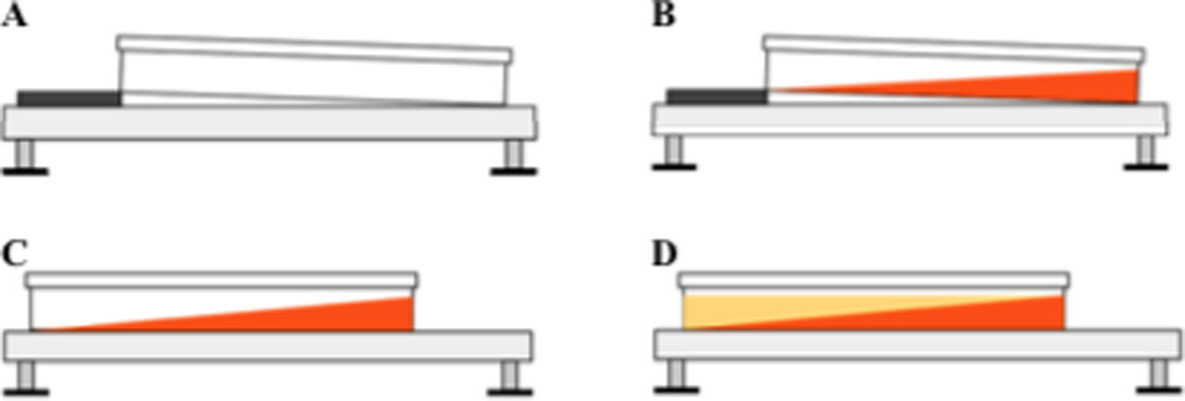
Schematic representation of the various steps of gradient plating. **A**: Original setting of plate in tilted position prior to pouring first media layer; **B**: Pouring of first media layer with high substrate concentration; **C**: Placement of plate in horizontal position after agar setting and prior to pouring second media layer; **D**: Pouring of second media layer on top of the first one

A recent study describes the successful development and application of these techniques to produce gel-stabilised gradient plates, which replicate naturally occurring environmental gradients and can be used to enrich and isolate microbial strains (More-Mutch et al. submitted). This method (briefly described in Fig. 6) is easily adapted to different types of environmental gradients, and retains the advantages of regular agar-based media while increasing their level of complexity. This new methodological approach is particularly useful in more accurate replication of environmental conditions in the lab and has been shown to be immediately applicable to reproducing salinity gradients associated with brine bodies, and easily combined with pH gradients (either independently or in co-variation with salinity gradients).

The on-going development of reactors specially designed for the simulation of different environmental conditions has seen significant improvements (e.g., Herschy et al. 2014) and is currently being tested for the simulation of chemical gradients in liquid media (see more details in Sect. 6.2). When this technology is further developed it should provide a major step forward in our capabilities to mimic the complexity of natural environments.

## Hydrothermal Vents

### Earth’s Hydrothermal Systems and Closed-System Hydrothermal Reactors

Since the discovery of deep-sea hydrothermal vents at Galapagos Spreading Center in 1977 (Corliss et al. 1979), ocean floor explorations have been carried out throughout Earth’s oceans. Through these explorations, hundreds of hydrothermal vent systems have been found near mid-ocean ridges, (back)arcs and hot spots (Beaulieu et al. 2015). As a result, it was revealed that composition of hydrothermal vent fluids varies depending on the geological setting, such as water depth, distance from the spreading centre and host rock composition (e.g., Tivey 2011). Meanwhile, ocean floor drilling provided successive depth profiles of mineralogical and geochemical data of hydrothermally altered crusts, which revealed thermal structure and elemental migration processes in subseafloor hydrothermal systems (e.g., Alt 2013). With the progress of the explorations of natural hydrothermal systems, experimental/thermodynamic simulations of water-rock reactions have also largely contributed to understanding of mechanisms that generate compositional variations of hydrothermal vent fluids (e.g., Seyfried and Mottl 1982; Seyfried and Ding 1995).

**Fig. 7:**
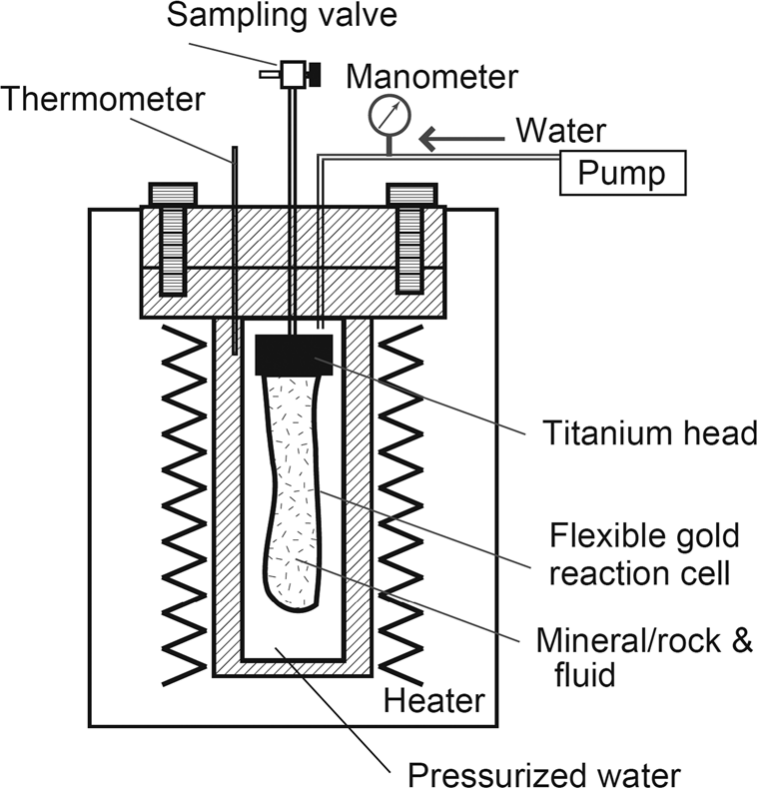
A schematic illustration of a Dickson-type closed-system reactor used in experimental simulations of hydrothermal systems on early Earth and Enceladus (Yoshizaki et al. 2009; Shibuya et al. 2013; Hsu et al. 2015; Sekine et al. 2015; Shibuya et al. 2015; Ueda et al. 2016) (modified from Yoshizaki et al. 2009). During sampling of a fluid, pressurised water is introduced into the reactor using a pump, compressing a flexible gold reaction cell. This enables to collect a fluid sample in the reaction cell via a sampling value without a significant change in pressure and temperature. The on-line sampling system allows to obtain variations in concentrations of dissolved species during experiments

The experiments have been mainly conducted with Dickson-type hydrothermal reactors for reproducing high-temperature and -pressure conditions of hydrothermal systems (Dickson et al. 1963; Seyfried et al. 1979). This type of reactor can achieve high pressures and temperatures, typically up to ca. 50 MPa and ca. 773 K, and can be used for reproducing closed-system water-rock reactions and has been widely applied to simulations of mafic- and ultramafic-hosted hydrothermal systems (Seewald and Seyfried 1990; Seyfried et al. 2007). Dickson-type reactors together with a flexible gold reaction cell allow to perform on-line sampling of fluids without significant changes in pressure and temperature conditions (e.g., Seewald and Seyfried 1990 (Fig. 7)). Decades of experimental work in this area have revealed that major inorganic compositions (pH and major elements) of high-temperature hydrothermal fluids (e.g., from black smoker vents) are mostly governed by mineral-fluid equilibria and additional magmatic inputs near the root zone of the hydrothermal circulation cell (Seyfried et al. 1991). Among the water-rock reaction processes, H_2_ generation by serpentinisation of ultramafic rocks has attracted much attention because low-biomass, archaeally dominated microbial ecosystems are found in H_2_-rich hydrothermal vent systems, utilising abiotically generated formate (e.g., Kelley et al. 2001, 2005; Takai et al. 2004; Lang et al. 2018). Generally, ultramafic-hosted hydrothermal systems generate H_2_-rich (i.e., high levels of free energy) hydrothermal fluids (H_2_$\mbox{concentration} = 1\mbox{--}20~\mbox{mmol}\,\mbox{kg}^{-1}$) (Charlou et al. 2002; Gallant and Von Damm 2006; Kumagai et al. 2008; Proskurowski et al. 2006), which is approximately one to two orders of magnitude higher than those of basalt-hosted systems (e.g., Charlou et al. 2002). This high H_2_-generation potential of ultramafic rocks and its reaction kinetics have been also experimentally examined (Allen and Seyfried 2003; Seyfried et al. 2007; Mayhew et al. 2013; McCollom et al. 2016).

Due to the extremely reducing conditions of ultramafic-hosted hydrothermal systems, the hydrothermal vent fluids are generally enriched in hydrocarbons and other organic compounds, which can be sourced either abiotically or biotically (e.g., Konn et al. 2009; Proskurowski et al. 2008). In particular, CH_4_ as well as sulphate/sulphur are considered as important energy sources for methane-oxidising, methanogenic, sulphur-oxidising, and sulphate-reducing microorganisms in the vent systems (e.g., Kelley et al. 2001, 2005; Brazelton et al. 2006). Experimental studies have revealed that the formation and decomposition of organic molecules are largely controlled by kinetic reactions under hydrothermal conditions (Seewald et al. 2006; McCollom and Seewald 2001, 2003a,b). More importantly, it was demonstrated that CH_4_ formation from CO_2_ in the hydrothermal system is kinetically limited even though CH_4_ is thermodynamically more stable than CO_2_ under H_2_-rich conditions (e.g., McCollom 2016). Therefore, abiotic CH_4_ production by CO_2_ reduction is probably limited in natural hydrothermal systems. Instead, a recent geochemical study of CH_4_-bearing natural hydrothermal fluids suggested that CH_4_ in the vent fluid was derived from fluid inclusions in plutonic rocks, where abiotic reactions with magmatic volatiles had occurred at temperatures higher than 673 K (McDermott et al. 2015).

In the past decade, Dickson-type closed-system reactors have been also used to simulate seafloor hydrothermal systems on early Earth (e.g., Yoshizaki et al. 2009) because H_2_-rich, alkaline hydrothermal vents on the seafloor have been proposed as one of the most probable birthplaces of life (e.g., Russell and Hall 1997; Russell et al. 2014; Martin and Russell 2007; Sojo et al. 2016). Moreover, hydrothermal alterations of Archean oceanic basalts and komatiites are distinctively different from modern equivalents (Nakamura and Kato 2004; Rouchon and Orberger 2008; Shibuya et al. 2012). Experimental studies have revealed that high concentrations of CO_2_ and thus lower pH of early Earth’s oceans (Halevy and Bachan 2017) had the potential to elevate *in situ* pH and reduce metal concentrations in hydrothermal fluids even at high temperatures, in contrast to their modern equivalents (Shibuya et al. 2013). Additionally, the experimental studies show that H_2_ concentrations of hydrothermal fluids in komatiite-hosted systems would be comparable to those in modern ultramafic-hosted hydrothermal systems (Shibuya et al. 2015; Ueda et al. 2016). These experimental studies suggest the existence of H_2_-rich alkaline hydrothermal vents on early Earth (Macleod et al. 1994). Laboratory experiments using closed-system reactors provide key information for reconstructing hydrothermal environments that cannot be seen on modern Earth.

### Chemical Evolution on Early Earth and Flow-System Experimental Simulations

**Fig. 8:**
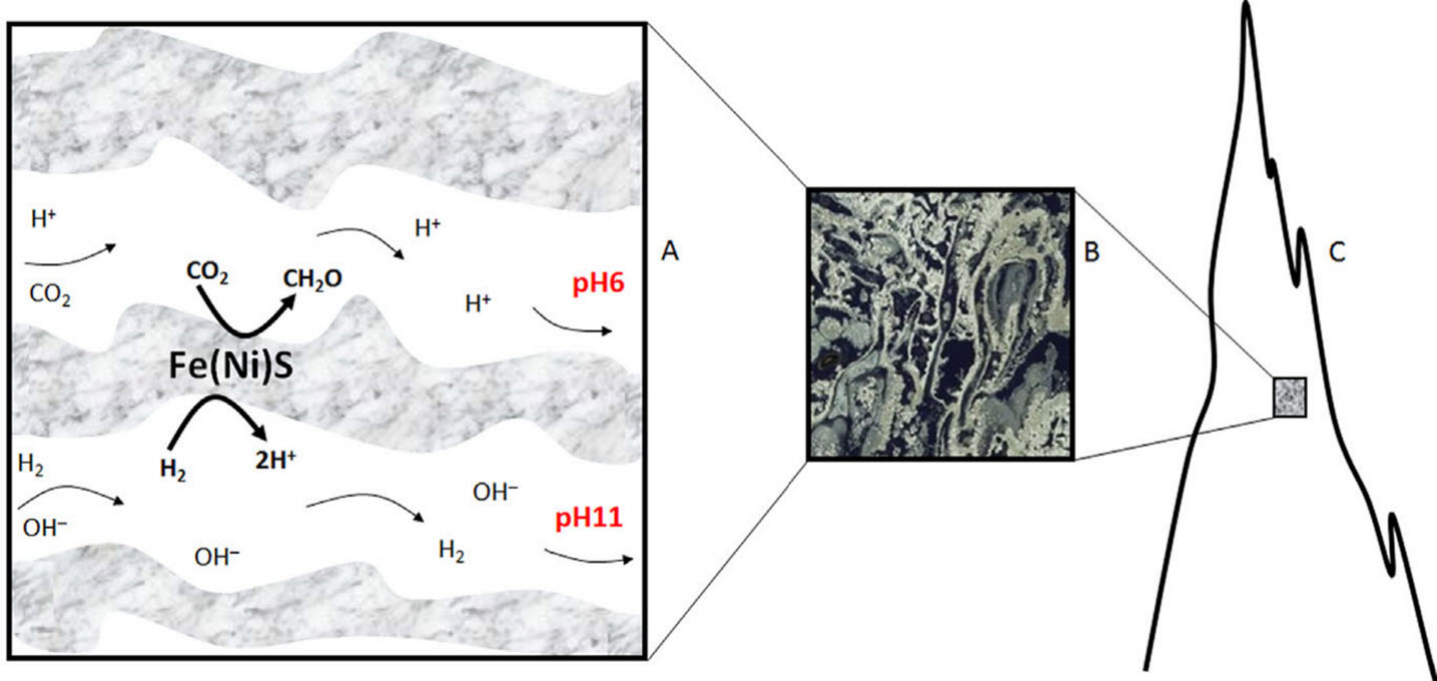
Reduction of CO_2_ by H_2_ across Fe(Ni)S barriers within an alkaline hydrothermal vent. (**A**) Electrons can theoretically be transferred across semiconducting barriers containing Fe(Ni)S minerals from H_2_ in alkaline hydrothermal solutions to CO_2_ in relatively acidic ocean waters to form organics. (**B**–**C**) Organics formed would not get lost to the ocean as the microporous structure (Deborah Kelley, University of Washington) could foster their accumulation due to thermophoresis. Recast from Lane (2017)

In addition to a supply of high levels of H_2_ (i.e., free energy, see Sect. 6.1), alkaline hydrothermal vents on early Earth could have also acted as the “cradle” of life on Earth through promoting prebiotic chemistry (e.g., Russell et al. 2014; Martin and Russell 2007; Sojo et al. 2016). One of the key features of early Earth’s alkaline hydrothermal vents, which suggest they played a role during abiogenesis, is the presence of natural pH and redox gradients across Fe(Ni)S and Fe-oxyhydroxide barriers precipitated in hydrothermal chimneys (Lane 2017). The proposed properties of such hydrothermal systems have been extensively studied by Russell and colleagues (Martin and Russell 2007; Martin et al. 2008; Russell and Hall 1997; Russell et al. 2014, 1994), suffices to say here that such natural pH and redox gradients across early Earth’s hydrothermal chimneys would have been the result of the mixing of warm (ca. 343–373 K), reducing sulphide-containing and H_2_-rich hydrothermal effluents with cooler, mildly acidic Fe^2+^ and CO_2_-rich oceanic waters. It has been hypothesised that due to the pH-dependant modulation of reduction potential when protons are involved in redox reactions such steep pH gradients across Fe(Ni)S inorganic barriers could have promoted the non-enzymatic reduction of CO_2_ into organic molecules (e.g. formate or formaldehyde) with electrons from H_2_ (Fig. 8) (Yamaguchi et al. 2014; Lane 2017).

**Fig. 9:**
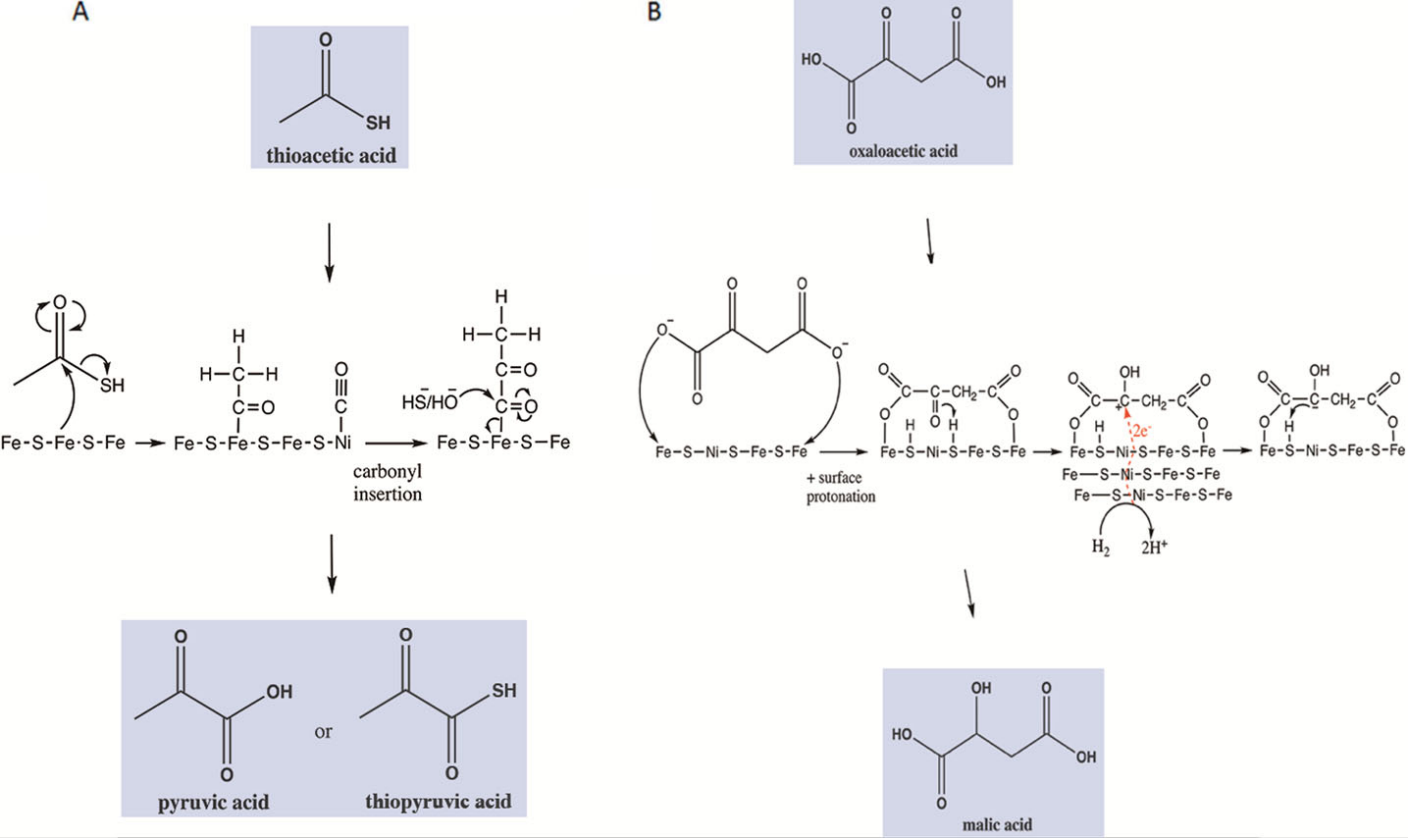
The reactivity of the Fe(Ni)S surfaces of Hadean alkaline hydrothermal vents could have promoted reactions analogous to those of the acetyl CoA pathway and incomplete reverse Krebs cycle. (**A**) Carbonylation of a bound acetyl group by bound CO (from CO_2_ reduction), followed by elution through nucleophilic attack by a sulphydryl or hydroxyl ion yielding thiopyruvic acid or pyruvic acid, respectively. (**B**) Reversible adsorption of oxaloacetate followed by hydrogenation of its keto group by two electrons (from H_2_ oxidation by Ni^2+^ in the alkaline phase) and H^+^ ions from surface mineral protonation yielding malic acid. Nickel atoms channel electrons from H_2_ catalysing a two-electron reduction. Recast from Camprubi et al. (2017)

Analogous conditions to those described above could, in principle, allow for further transformations (mostly carbonylations and hydrogenations) yielding non-enzymatic versions of the acetyl CoA pathway and incomplete reverse Krebs cycle (Fig. 9) (Camprubi et al. 2017). Both proto-metabolic pathways would have been of great abiogenic importance since a wide plethora of relevant biomolecules (amino acids, lipids, nucleotides and cofactors) can be derived from their intermediates. Thus, establishing under which precise experimental conditions such reactions are (and are not) promoted is of paramount interest to this field of research.

Some experimental effort has gone into testing the aforementioned predictions. For instance a large open-flow reactor simulating silica-rich hydrothermal percolations on early Earth (Herschy et al. 2014) was successful at synthesising fairly crystalline mackinawite—a biologically relevant FeS mineral formed from the precipitation of Fe^2+^ and HS^−^ (Rickard et al. 2006; White et al. 2015) whose atomic structure is found in enzymes such as some hydrogenases (McGlynn et al. 2009; Nitschke et al. 2013; Nitschke and Russell 2013)—and also allowed for the detection of μM quantities of formaldehyde. Unfortunately, this approach did not afford highly reproducible results in this regard, most probably due to the large stochasticity of the fine experimental conditions associated to large-scale reactors.

In order to address these issues, more recent work has focused on developing microfluidics devices (Möller et al. 2017). Microfluidics or the usage of small (often μL) reaction volumes is a good experimental approach when a strict control of experimental reactions (over time and space) is required. It has been shown that microfluidics devices offer great control when simulating steep pH gradients generated due to laminar co-flow of solutions at different pH (i.e. the simulated gradients do not dissipate even when both solutions are in contact) (Möller et al. 2017). On another hand, electrochemical studies—some employing a microfluidics approach—have been largely successful at promoting non-enzymatic redox reactions (Barge et al. 2018), including CO_2_ reduction (Qiao et al. 2014). Despite this, such combination is now starting to emerge as a way of testing for CO_2_ reduction in an abiogenic scenario.

At Utrecht University, a microfluidics device is developed, which aims to simulate the synthesis of organics at AHVs on early Earth. This approach offers the advantages mentioned above associated with microfluidics but will also (i) allow for high pressure (up to 20 MPa) open-flow simulations, a feature which has not been duly explored due to its technical complexity. High pressure is of crucial importance, not just because it offers a more realistic simulation of the conditions at the ocean floor, but also because at ambient pressure H_2_ is hardly soluble (Wiebe et al. 1932), an important problem when hypothesising H_2_ as a prebiotic source of reducing power. Furthermore, this new device aims to (ii) allow for a fine control over the usage of the reaction catalysts (Fe(Ni)S minerals such as mackinawite, greigite and violarite). In previous setups the precipitation of the catalytic minerals was performed *in situ* (i.e. simultaneous to the synthesis of organics). Needless to say, such an approach reflects more closely the natural conditions found at real hydrothermal systems, but also heavily limits the assessment of the impact of the mineral characteristics (e.g. crystallinity, thickness, lattice orientation, etc.) over the overall simulation.

In summary, this new approach builds upon the previous work and aims to finely understand the effect of each experimental variable, with a clear focus on high pressure simulations and a detailed characterisation of the catalysis process. Its main goal is to better determine whether natural pH gradients could have acted as primordial sources of free energy and thus fostered the emergence of life on Earth.

### Hydrothermal Activities Beyond Earth and Their Experimental Simulations

Given the widespread existence of H_2_O, rocks, and heat in planetary bodies, hydrothermal environments are expected to be ubiquitous in the Solar System (e.g., Vance et al. 2016; Neveu et al. 2017). In fact, recent spacecraft explorations have found geochemical evidence for the occurrence of hydrothermal activities on Mars and Enceladus (e.g., Squyres et al. 2008; Hsu et al. 2015; Berger et al. 2017; Waite et al. 2017).

Geologically-active icy satellites, such as Europa and Enceladus, orbiting around the gas giants are known to possess subsurface oceans that interact with the rocky seafloor (e.g., McKinnon 2015; Vance et al. 2016). Due to tidal and long-lived radiogenic heating within the rocky core, high-temperature fluids could be formed within pores of the rocks and would erupt into the cold subsurface ocean, generating hydrothermal vent systems at the interface between the ocean and rocky core (e.g., Vance et al. 2007, 2016; Choblet et al. 2017). A “smoking gun” of such a hydrothermal activity within an icy moon is the finding of a hydrothermal product of nanosilica particles and H_2_ in the plume (Hsu et al. 2015; Waite et al. 2017). The hydrothermal activity within Enceladus also could play a role for the formation of complex organic matter found in the plume (Postberg et al. 2018b).

Mars is another planetary body that possessed extensive hydrothermal environments in the past. Mars orbiters have found the abundance of phyllosilicates (e.g., chlorite, serpentine, and smectite) formed by water-rock reactions (e.g., Buczkowski et al. 2010; Ehlmann et al. 2011; Poulet et al. 2005). These crustal clay minerals would have been generated by alterations of the primordial crust by a steam atmosphere immediately after the formation (Cannon et al. 2017) and/or by interactions with groundwaters in later stages (Ehlmann et al. 2011). Given a high heat flux on early Mars and high porosity of Martian crust, a substantial amount of groundwaters would have existed within deep crust, plausibly causing water-rock interactions at high temperatures on early Mars (Michalski et al. 2013). The NASA Mars Exploration Rover Spirit and the Mars Science Laboratory Rover Curiosity have found geochemical evidence for upwelling of hydrothermal fluids and Ge and Zn enrichments (Squyres et al. 2008; Berger et al. 2017), within sediments near their landing sites.

Given the presence of mafic- and ultramafic rocks on these bodies (Michalski et al. 2013; Sekine et al. 2015; Choblet et al. 2017), H_2_-rich alkaline hydrothermal fluids may have been generated via hydrothermal activities, similar to alkaline hydrothermal vents on early Earth (also see Sect. 6.1). Within Enceladus, the reducing-alkaline fluids would be upwelling into cold CO_2_-rich waters of the subsurface ocean (Waite et al. 2017; Choblet et al. 2017). On early Mars, upwelled reducing groundwater would have interacted with near-surface oxidants and atmospheric CO_2_ on the surface (e.g., Michalski et al. 2013; Hurowitz et al. 2017). These redox interactions could have provided free energy for prebiotic chemistry and early life (also see Sect. 6.2). In addition, reducing gas species, such as H_2_, provided by hydrothermal reactions would be essential to keep early Mars warm (Ramirez et al. 2014; Wordsworth et al. 2017).

Icy dwarf planets, Ceres and Pluto, are also suggested to have possessed hydrothermal activities in their early history (e.g., Castillo-Rogez and McCord 2010; Neveu and Desch 2015; Neveu et al. 2017; Sekine et al. 2017). Ceres’ interior would have become high temperatures (e.g., ca. 573 K) in the first 1 billion years after its formation (e.g., Castillo-Rogez and McCord 2010; Neveu and Desch 2015; McCord and Sotin 2005). The occurrence of such high-temperature water-rock interactions is evidenced by the widespread occurrence of serpentine, smectite, carbonate, and aliphatic organic matter on the surface (De Sanctis et al. 2015, 2017). Pluto may have also experienced melting of ice in the early history (Sekine et al. 2017). Upon the Charon-forming impact, Pluto’s equatorial regions have been heated up to ca. 373 K (Sekine et al. 2017).

The volatile compositions of these dwarf planets are critical to constrain the occurrence of migration of protoplanets in the disk (e.g., Walsh et al. 2011; De Sanctis et al. 2015). Knowledge on hydrothermal reactions occurred on these bodies is essential to reconstruct the primordial volatile compositions based on the surface materials.

Despite of such a variety of importance, only a few experimental studies, thus far, have been performed to simulate hydrothermal reactions beyond Earth (for Enceladus, see Hsu et al. 2015; Sekine et al. 2015; Barge and White 2017). Laboratory simulations of these icy bodies need to achieve the expected high-pressure conditions on planets/satellites. Due to the deep subsurface ocean, Europa’s water-rock reactions would proceed at high pressures of 100–200 MPa (Vance et al. 2016). Ganymede and Titan may have possessed the hot water mantles during the accretion, where water-rock interactions occurred at 1000–2000 MPa (e.g., Kuramoto and Matsui 1994). The pressure conditions of hydrothermal reactions on early Mars would reach ca. 100–500 MPa (Vance et al. 2016). Compared with the pressure conditions of hydrothermal vents on Earth (ca. 20–50 MPa) and Enceladus (ca. 10–50 MPa), the proposed pressures for large icy moons and early Mars are high. An increase in pressure could affect kinetics and mechanisms of hydrothermal reactions through an increase in collision number of reactants and a change in thermochemical stability of intermediate/final products. Existing Dickson-type closed-system reactors and open-flow system simulators typically achieve pressures up to 20–50 MPa (see Sects. 6.1 and 6.2); thereby, further technical advances would be required. Given high gravity and, thus, high pressures at seafloors on habitable super-Earths, high-pressure hydrothermal experiments would be also important to predict geochemical cycles occurring there.

Laboratory simulations of these icy bodies also need to use rock analogues that have similar mineralogical and chemical compositions to the rock components. Similar to laboratory simulations of hydrothermal reactions on early Earth (Yoshizaki et al. 2009; Mielke et al. 2010; Shibuya et al. 2013), synthesised analogue rocks of Martian crust are used to simulate hydrothermal environments on early Mars (Tosca et al. 2004). A usage of synthesised analogue of chondrites may be important to simulate the ocean worlds in icy moons (Barge et al. 2018).

## Interior Structure

Computer-based (*in silico*) simulations can help us to infer the composition and interior structure of icy moons based on different observational constraints. Equation of states (EoS) of different materials can be derived from *ab initio* calculations based on first principles in physics, or from laboratory data for Earth materials. EoS are needed to understand how the mass is distributed within an icy moon depending on its composition (in rough terms water, silicate rock and iron content). The mass distribution depends on the compressibility of the material with lithostatic pressure, expansion with temperature, melting (e.g., partially molten cores or silicate differentiation by rocky crust formation), and other factors such as light element addition to the core, hydration of the rocky layer, and temperature evolution of the entire body. On the other hand, the mass distribution is reflected in the moment of inertia factor, which can be measured from space. The so-called Love numbers describe a body’s rigidity and response to tidal potentials (from the Sun, planets or other moons in the system) and can be measured from orbit, delivering an additional constraint on the interior and ice crust thicknesses. The motion of a rotating body (for example via phase librations) can tell us about the existence of liquid material beneath the surface and crustal thickness (Van Hoolst et al. 2013). Other observational constraints on liquid layers include vapour plumes as have been detected for Enceladus and Europa or magnetic fields—either self-induced in a liquid core via a magnetic dynamo or induced in a subsurface ocean through interaction with a planet’s magnetic field.

### Induced Magnetic Fields

Some of the most convincing evidence for liquid water layers in extraterrestrial worlds comes from magnetic field signatures. The principle mechanism of the induction technique is that time-variable magnetic fields induce electric fields according to Faraday’s law. These electric fields can drive electric currents in electrically conductive layers and thus produce secondary induced magnetic fields. Observations of induced, i.e., secondary, magnetic fields near a planetary body therefore reveal the existence of an electrically conductive layer such as a saline subsurface ocean (Khurana et al. 1998; Neubauer 1998; Zimmer et al. 2000; Kivelson et al. 2000). The primary time-variable magnetic fields, i.e., the inducing fields, originate in case of Jupiter from the tilt of Jupiter’s dipole magnetic moment with respect to its spin axis by approximately 10 degrees. The resulting time-periodic inducing magnetic field in the rest-frame of the moons is the synodic rotation period of Jupiter (e.g., Saur et al. 2010; Seufert et al. 2011).

The amplitudes and phases of observed induced magnetic fields near the moons provide information about the conductivity structure of the interior. The Galileo spacecraft discovered induced magnetic fields near the icy satellites Europa, Ganymede and Callisto (Khurana et al. 1998; Zimmer et al. 2000; Kivelson et al. 2002). Because saline liquid water, but not frozen water, possesses an electrical conductivity sufficiently large to generate the measured induced fields, the Galileo magnetic field observations provide strong evidence for subsurface oceans. However, observations at only one inducing frequency, i.e. the synodic rotation period of Jupiter, are non-unique because the depth, the conductivity and the thickness of the layers cannot be resolved independently. Future observations by NASA’s Europa Clipper and ESA’s JUICE mission will measure at further inducing frequencies to better characterise the oceans.

Interpretation of magnetic field measurements near the moons requires modelling because the following magnetic field contributions next to induction in the interior need to be considered quantitatively: (1) The interaction of the magnetospheric plasma with the thin atmospheres of the moons can generate large magnetic fields. In case of Europa, this effect was considered and a stronger case for an ocean within Europa was derived (Schilling et al. 2007). (2) In case of Ganymede, the magnetic dynamo in its core makes the existence of induction signals from an ocean uncertain (Kivelson et al. 2002). However, Hubble Space Telescope observations of the oscillation of Ganymede’s auroral ovals are not subject of this ambiguity and could confirm Ganymede’s ocean (Saur et al. 2015). At Callisto, induction in its ionosphere plays an important role (Hartkorn and Saur 2017). Therefore, the existence of an ocean within Callisto is uncertain.

### High-Pressure Phases of Ice, Silicates and Metals

The water phase diagram is composed of a complex suite of polymorphs with distinctive physical and thermodynamic properties that have been studied with an uneven effort since the early work of Bridgman (1912, 1937). There are, to this day, 18 known solid phases (stable and metastable), along with vapour and liquid H_2_O, and other may still be discovered. A more detailed description of water phases and their properties is provided in Journaux et al. (2019).

For water worlds only a few stable phases are of direct relevance to the conditions found in their hydrosphere: liquid water, ice Ih, III, V and VI for icy moons of our Solar System (Sotin and Tobie 2004), and possibly ice VII and the ionic ice X for thicker water-rich exoplanets hydrosphere (Sotin et al. 2007). While many aspects of these phases affect the interior structure of ocean worlds, the most fundamental is that ice Ih is less dense than liquid water, while the higher phases sink in pure liquid water, but could potentially float in concentrated brines (Vance et al. 2014). The thermodynamic properties of liquid water were recently revised based on new experiments (Bollengier et al. 2019), which enables more accurate and self-consistent consideration of the phase relations and thermal transport in ocean worlds.

Ice Ih is currently best described by Feistel and Wagner (2006) representation up to 210 MPa and liquid H_2_O by the IAPWS formulation (Wagner and Pruß^2002^) up to 1 GPa. Ice III and V PVT-EoS have received little experimental and theoretical attention in the last decades, but recent effort have allowed to derive a Gibbs energy formulation of their EoS presented in more details later on in this book (Journaux et al. 2019). Ice VI and VII have been studied experimentally and theoretically with more details (Journaux et al. 2019; Klotz et al. 2017). Ice X remains poorly studied to date because of the challenges to achieve static high pressure experiments with water over 100 GPa. Current PV-EoS are based on *ab initio* data and do not have a thermal component (Journaux et al. 2014).

Solid-solid phase transitions also occur in the rocks that make up the mantle of larger satellites such as Ganymede. Based on the elements in the solar spectrum compared with the mineral composition in chondritic material (Beatty et al. 1999) and probes from other meteorites and Earth’s crust (McDonough and Sun 1995), we can assume that the rocky mantle of the icy satellites is dominated by oxygen, magnesium, iron, silicon, aluminium, calcium, and sodium (which make up 99% of Earth’s mantle minerals, McDonough and Sun 1995). Experimental data have been fitted to Earth’s main minerals made up from these elements (e.g., Stixrude and Lithgow-Bertelloni 2005, 2011; Holland and Powell 1998).

A putative metallic core in the deep interior of icy moons should be dominated by iron or nickel. Seismological observations of Earth’s core suggest the additional presence of light alloying elements (Birch 1952) such as for example sulphur, oxygen, carbon, silicon, and hydrogen (for a recent review, see Hirose et al. 2013). These elements are cosmochemically abundant (Lodders and Fegley 1998) and have therefore been postulated to also be present in the metallic cores of terrestrial planets and (icy) moons. Though a variety of core alloys are feasible, the most studied composition is the binary eutectic mixture of iron and sulphur (Fe-FeS). The solid phase is $\gamma $-Fe for iron-rich and FeS V for sulphur-rich Fe-FeS alloys in the pressure range relevant for the cores of icy moons ($<10~\mbox{GPa}$). The densities of $\gamma $-Fe (Tsujino et al. 2013) and FeS V (Urakawa et al. 2004) are reasonably well described by a third order Birch-Murnaghan equation of state that has been fitted to data from *in situ* synchrotron X-ray diffraction experiments. Densities of liquid Fe-FeS alloys have been extensively studied by using various techniques (Balog et al. 2003; Chen et al. 2014; Nishida et al. 2008; Sanloup et al. 2000; Nishida et al. 2011). The different data sets, however, do not always agree. A recent study employing *in situ* X-ray diffraction and *ab initio* calculations provides the hitherto most coherent data set for EOS of small planetary Fe-FeS cores (Morard et al. 2018). Melting temperatures have been extensively studied for primarily the end-members Fe, FeS as well as the eutectic concentration at pressures of small planetary cores (Usselman 1975; Fei et al. 1997, 2000; Li et al. 2001; Stewart et al. 2007; Chudinovskikh and Boehler 2007; Chen et al. 2008; Morard et al. 2007, 2008). Melting temperatures of the iron-rich side can be described by a thermodynamic model of binary liquid mixing (Buono and Walker 2011). The sulphur-rich side is lacking such a model and the liquidus as a function of sulphur concentration is thus determined by linearly interpolating between end-members (Rückriemen et al. 2018). Recent models indicate that core freezing in small planetary bodies such as icy moons may proceed from the top to the bottom instead of from the bottom to the top as assumed for Earth’s core (McKinnon 1996; Hauck et al. 2006; Rückriemen et al. 2015, 2018). Top-down freezing can have interesting implications on magnetic field generation and core structure as discussed in e.g. Zhan and Schubert (2012), Christensen (2015), Rückriemen et al. (2018) and Journaux et al. (2019).

### Interior Structure Models

An interior structure model gives a first idea on the distribution of material (here metals, silicate rocks and water or ice) within a planet or moon. Such a model uses information on mass and radius (hence density), and other possible constraints such as the existence of liquid water, compositional constraints, and orbital information to identify the thickness of the different material layers (as well as if they are differentiated, e.g. if silicate rocks and metals separated into two different layers or not). An interior structure model can therefore help to identify the extent of liquid subsurface oceans, or the possible existence of a metal core (with implications for a magnetic dynamo as observed for Ganymede).

For the larger icy moons of the Solar System we can assume hydrostatic equilibrium, which means that the external (gravity) force is in balance with the internal (pressure-gradient) force, leading to a rounded shape of the moons. As an approximation, we can therefore assume a spherical model, where averaged profiles of planetary properties vary only with depth (Schubert et al. 2001). The mass for example can be integrated over the planet depending on radius $r$ and density $\rho $:

1 $$ \frac{dm}{dr} = 4 \pi r^{2} \rho $$

For a planet of uniform density, the mass at any radius would be $m(r) = \frac{4}{3} \pi r^{3} \rho $. However, the density strongly varies with depth in bodies such as planets or moons, due to variations in composition (e.g. icy crust, liquid water ocean, silicate mantle, metal core) and due to compressibility effects. The temperature further influences the density of materials, where the strongest influence on density can be seen for water and ice, whereas the density of the metal core only weakly depends on the assumed temperature. While the temperature profile from core to surface depends on the material properties and heat exchange between the different layers, the gravitational acceleration and the pressure can be determined more easily. The gravitational force from centre to surface can be described by a differential equation named Poisson equation:

2 $$ \frac{dg}{dr} = \frac{d}{dr} \biggl( \frac{Gm}{r^{2}}\biggr) = 4 \pi G \rho - 2 \frac{g}{r} $$

The change in gravitational acceleration with radius $r$ depends also on the density $\rho $. $G$ is the gravitational constant. The formulation on the right side of Eq. (2) can be directly obtained from the derivative of the mass over $r$ from Eq. (1) and re-substituting that the gravitational acceleration at any radius equals $gm/r^{2}$. This reformulation allows to solve the Poisson equation without the need to solve the mass equation, therefore reducing the complexity of the problem.

The pressure $p$ evolves following the equation of hydrostatic equilibrium:

3 $$ \frac{dp}{dr} = - g \rho $$

For small variations in density and gravitational acceleration (for example close to the surface of the body), the pressure at depth $z$ can be approximated with $p = g \rho z$.

An interior structure model solves Eqs. (1)–(3) to investigate radii of the different layers—for icy moons from core to icy crust—for bodies under hydrostatic equilibrium (e.g., Wagner et al. 2011; Noack et al. 2016; Vance et al. 2018). For that, the density is either set to a fixed value for each layer, or radius-dependent densities are calculated using EoS. An EoS links the density (or volume) to temperature and pressure for any given material. They are based on the fundamental physical relationship between isothermal bulk modulus, volume and pressure (Murnaghan 1967), and are fitted to experimental or *ab initio*, theoretically derived data. Based on fundamental thermodynamic relations, all thermodynamic potentials are related to one another and can be derived from the Gibbs and Helmholtz free energy. By using an EoS to determine density variations with pressure and temperature for different materials, other thermodynamic properties can be directly calculated as derivatives from the Gibbs free energy (Poirier 2000). Similar to the equations listed for the interior structure model above, the moment of inertia (MOI) factor can be calculated based on the density variation in the interior and can be used to identify how mass is distributed in the interior. For a planet with uniform density, the MOI factor would be 0.4. Smaller values indicate a mass concentration in the centre of the body. For example, for a rocky body with a metal core extending to half the radius, and with a metal core density twice the silicate mantle density, the MOI factor would be 0.389. A fully differentiated body (metal core, rocky mantle, ice/water layer) containing large amounts of water such a Ganymede has a very low MOI factor just above 0.3, whereas Titan, of similar mass and radius, shows a much larger value (0.34) indicating incomplete differentiation (Iess et al. 2010; Schubert et al. 2004).

### Interior Dynamics

The interiors of icy satellites can have two low viscosity regions—iron alloy cores and liquid water oceans—that are unstable to fluid motions driven by thermocompositional convection and/or mechanical stirring (e.g., Christensen 2015; Soderlund et al. 2014; Noir et al. 2009; Soderlund 2019; Journaux et al. 2019). These fluid motions are governed by the continuity equation that describes the conservation of mass, the Navier-Stokes equation that describes the conservation of momentum, the heat and mass transport equations, and the induction equation (e.g., Braginsky and Roberts 1995). The continuity equation states that the mass flowing into and out of a control volume is equal to the rate of mass change within it:

4 $$ \frac{\partial \rho }{\partial t} + \boldsymbol{\nabla } \cdot ( \rho \mathbf{u} ) = 0 $$

where $\rho $ is density [$\mbox{kg}\,\mbox{m}^{-3}$], $t$ is time [s], $\boldsymbol{\nabla }$ is the spatial derivative operator [$\mbox{m}^{-1}$], and $\mathbf{u}$ is the velocity vector [$\mbox{m}\,\mbox{s}^{-1}$]. For incompressible fluids, (4) simplifies to $\boldsymbol{\nabla } \cdot \mathbf{u} = 0 $. The Navier-Stokes equation can be written in the rotating frame as:

5 $$\begin{aligned} &\rho \biggl(\frac{\partial \mathbf{u}}{\partial t} + \mathbf{u} \cdot \boldsymbol{\nabla } \mathbf{u} + 2 \boldsymbol{\varOmega } \times \mathbf{u} + \boldsymbol{\varOmega } \times (\boldsymbol{\varOmega } \times \mathbf{r} ) + \frac{\partial \boldsymbol{\varOmega }}{\partial t} \times \mathbf{r}\biggr) \\ &\quad = - \boldsymbol{\nabla } P + \rho \mathbf{g} + \mu \boldsymbol{\nabla}^{2} \mathbf{u} + \frac{1}{3} \mu \boldsymbol{\nabla } ( \boldsymbol{\nabla} \cdot \mathbf{u} ) + \mathbf{j} \times \mathbf{B} \end{aligned}$$

where $\varOmega $ is angular velocity of the system [$\mbox{s}^{-1}$], $P$ is pressure [$\mbox{kg}\,\mbox{m}^{-1}\,\mbox{s}^{-2}$], $\mathbf{g}$ is gravitational acceleration [$\mbox{m}\,\mbox{s}^{-2}$], $\mu $ is dynamic viscosity [$\mbox{kg}\,\mbox{m}^{-1}\,\mbox{s}^{-1}$], $\mathbf{j}$ is the current density vector [$\mbox{A}\,\mbox{m}^{-2}$], and $\mathbf{B}$ is the magnetic induction vector [$\mbox{kg}\,\mbox{s}^{-2}\,\mbox{A}^{-1}$]. Here, the first term is fluid acceleration, the second term is advection of the fluid, the third term is the Coriolis force, the fourth term is the centrifugal force, the fifth term is the temporal change of the system’s angular velocity, the sixth term is the pressure gradient, the seventh term is gravitational acceleration, the eighth and ninth terms are viscous dissipation, and the tenth term is the Lorentz force.

The heat and mass transport equations are given, respectively, by

6 $$\begin{aligned} \rho T \biggl( \frac{\partial S}{\partial t} + \mathbf{u} \cdot \boldsymbol{ \nabla } S \biggr) =& \boldsymbol{\nabla } \cdot (k \boldsymbol{\nabla } T ) + H \rho \end{aligned}$$

7 $$\begin{aligned} \rho \biggl( \frac{\partial \varXi }{\partial t} + \mathbf{u} \cdot \boldsymbol{\nabla } \varXi \biggr) =& \boldsymbol{\nabla } \cdot \bigl(k^{\xi } \boldsymbol{\nabla } \varXi \bigr) + H^{\xi } \rho \end{aligned}$$

where $T$ is temperature [K], $S$ is specific entropy [$\mbox{J}\,\mbox{kg}^{-1}\,\mbox{K}^{-1}$], $k$ is thermal conductivity [$\mbox{W}\,\mbox{m}^{-1}\,\mbox{K}^{-1}$], $H$ is internal heating [$\mbox{W}\,\mbox{kg}^{-1}$], $\varXi $ is mass fraction of the impurity concentration (e.g., sulphur in the core, salt in the ocean), $k^{\xi }$ is mass diffusion coefficient [$\mbox{kg}\,\mbox{m}^{-1}\,\mbox{s}^{-1}$] and $H^{\xi }$ is volumetric compositional sources [$\mbox{s}^{-1}$]. The left side of the equations represent the material derivative, while the right side represents diffusion and any non-conservative sources and sinks.

The magnetic induction equation is

8 $$ \frac{\partial \mathbf{B}}{\partial t} = \boldsymbol{\nabla } \times ( \mathbf{u} \times \mathbf{B} ) - \boldsymbol{\nabla } \times ( \eta \boldsymbol{\nabla } \times \mathbf{B} ) $$

where $\eta $ is magnetic diffusivity [$\mbox{m}^{2}\,\mbox{s}^{-1}$]. The first term is temporal evolution of the magnetic field, the second term represents magnetic induction, and the third term represents magnetic diffusion.

The heat equation (6) as well as the continuity equation (4) and the Navier-Stokes equation (5) also hold in high-viscous layers such as the rocky mantle and the ice layers. Terms referring to the magnetic induction or angular velocity can here be neglected. In addition, the first two terms in Eq. (5) are negligibly small for high viscosities and are considered to be zero under the so-called Infinite-Prandtl-number assumption, where the Prandtl number is a non-dimensional parameter describing the ratio of kinematic viscosity (momentum diffusivity) over thermal diffusivity.

Heat sources in the energy equation (6) can include internal heating by radioactive decay or tidal heating, gravitational heat release inside the core due to chemical convection (where lighter elements float upwards and the heavier iron sinks downwards), and latent heating due to solidification of iron in the core and/or melt in the mantle. Depending on the interior temperature, the silicate core of icy bodies may experience convection of rocky material over time, similar to the larger rocky bodies in the Solar System such as Earth. Convection would allow more heat to be released into the ocean or ice above the silicate shell, allowing for increased hydrothermal vent activity or even partial melting of the hydrated rocks.

When modelling convection in small bodies (in both the silicate core and in the ocean-ice shell), where compressional effects acting on thermodynamic parameters such as density are negligible, the numerical treatment of the conservation equations often employ the common approximation of a Boussinesq fluid, where the fluid properties are held constant except for density anomalies in the gravity term in Eq. (5). Buoyancy effects are maintained as the density is assumed to have mean and fluctuating components associated with thermal and compositional anomalies. In shallow oceans with depths much smaller than the satellite radius, the equations may be further simplified to the so-called primitive equations if vertical flows are assumed to be much weaker than horizontal flows (e.g., Marshall et al. 1997).

A key consideration for simulations of turbulent geophysical fluids is that numerical models are unable to resolve the fine-scale turbulence that dissipates kinetic energy in fluids with low viscosity (high Reynolds number), and must use a viscosity several orders of magnitude higher than the real world. Strategies for dealing with this include nondimensional rescaling and eddy closure schemes (Pope 2000). This is a problem for atmospheres, oceans, and liquid cores everywhere, but is especially problematic in icy world oceans where observational constraints on flow are not yet available.

## Conclusion

The discovery and characterization of subsurface oceans on icy moons, especially on Enceladus and Europa, have expanded our understanding of habitability in our Solar System. Before these findings, extraterrestrial life was expected to be found only on planetary bodies within the classical habitable zone (Kasting et al. 1993). Robotic missions like NASA’s Cassini and Galileo collected an assortment of data which has to be interpreted using information gained by *in situ*, *in silico*, and *in vitro* Earth-based experiments.

These Earth-based experiments are for example performed using light gas guns to simulate the ejection of material or sample collections. Further, the calibration of in situ mass spectrometers for the analysis of ice grains emitted from subsurface ocean into a plume with a laser-driven LILBID analog experiment, is a very promising approach. Currently a spectrum library with characteristic organic spectral signatures of biotic (e.g., peptides) and abiotic origin is in the making to enable future missions to safely distinguish between the two cases. Future experiments should include samples of real biogenic material from e.g. viruses, bacteria, archaea and small eukaryotes.

Exploring and analysing the environmental conditions and the behaviour of microorganisms in icy moons analogue field sites on Earth is another important step towards a better understanding of the habitability of these icy worlds. These studies should be accompanied by lab-based experiments simulating the potential habitable areas on/in an icy moon and investigating the capability of terrestrial organisms to survive and multiply under these conditions. However, simulations of physically more extreme conditions also must be performed. For that, further approaches in material properties under conditions occurring in icy worlds have to be made in connection with studies about the thermodynamics of aqueous solutions. A main focus should also lie on experiments reproducing high-temperature and -pressure conditions of hydrothermal systems. The aforementioned studies have to be completed by simulation experiments directly in space, which are planned for gaining insight into the potential of the stability of organics, biomolecules and microorganisms and about their potential to be detectable after being exposed to extreme space conditions.

Despite on-going efforts to better recreate complex, gradient-rich environments in the lab (both in solid and liquid media), a stronger emphasis on new developments, improvements, and widening of their use are clearly necessary.

The importance of parallel *in silico* experiments should not be underestimated. Computer based models about the interior structure, the magnetic field, and the interior dynamics are necessary for a broad understanding of the processes in and on an icy moon, and should be adapted to incorporate latest experimental insights.

These experiments will also help to configure the instruments on future space missions to the outer Solar System. Linking techniques, models, and experimental designs provided by scientific fields that include astronomy, physics, geochemistry, and microbiology is essential to fulfilling these tasks. Current experimental and simulation efforts in the field of ocean world research span a range of disciplines. By bringing these traditionally disparate topics together, planetary science and astrobiology promise to provide new insight. This review is meant to catalyse further transdisciplinary cooperation: we will need to join forces to reveal the mysteries of these spectacular ocean worlds.
